# Changes in Sexual Behaviors with Opposite-Sex Partners and Sexually Transmitted Infection Outcomes Among Females and Males Ages 15–44 Years in the USA: National Survey of Family Growth, 2008–2019

**DOI:** 10.1007/s10508-022-02485-3

**Published:** 2022-12-06

**Authors:** David A. Katz, Casey E. Copen, Laura T. Haderxhanaj, Matthew Hogben, Steven M. Goodreau, Ian H. Spicknall, Deven T. Hamilton

**Affiliations:** 1 Department of Global Health, University of Washington, Box 351620, Seattle, WA 98195 USA; 2 Division of STD Prevention, National Center for HIV/AIDS, Hepatitis, Sexually Transmitted Disease, and Tuberculosis Prevention; Centers for Disease Control and Prevention, Atlanta, GA USA; 3 Center for Studies in Demography and Ecology, University of Washington, Seattle, WA USA; 4 Department of Anthropology, University of Washington, Seattle, WA USA

**Keywords:** Sexual behavior, Screening, Sexually transmitted infections, Sexual networks

## Abstract

Rates of reported gonorrhea and chlamydial infections have increased substantially over the past decade in the USA and disparities persist across age and race/ethnicity. We aimed to understand potential changes in sexual behaviors, sexual network attributes, and sexually transmitted infection (STI) screening that may be contributing to these trends. We analyzed data from 29,423 female and 24,605 male respondents ages 15–44 years from the National Survey of Family Growth, 2008–2019. We used survey-weighted linear or logistic regression to evaluate linear temporal trends in sexual behaviors with opposite-sex partners, network attributes, and STI testing, treatment, and diagnosis. Significant declines were observed in condom use at last vaginal sex, mean number of vaginal sex acts, proportion of condom-protected sex acts in the past 4 weeks, and racial/ethnic homophily with current partners among males and females from 2008–2010 through 2017–2019. Among males, mean number of female partners in the past 12 months and concurrency also declined, while the percent reporting ever having sex with another male increased. Past-year testing for chlamydia and any STI increased among females. Research is needed to understand how these changes interact and potentially contribute to increasing reported gonorrhea and chlamydia diagnoses and identify avenues for future intervention.

**Supplementary Information:**

The online version contains supplementary material available at 10.1007/s10508-022-02485-3.

## Introduction

Rates of reported gonorrhea and chlamydial infections have increased substantially over the past decade in the USA (Centers for Disease Control & Prevention, 2021) The Centers for Disease Control and Prevention estimated that new infections in 2018 alone resulted in nearly $1 billion in lifetime direct medical costs (Chesson et al., 2021). Although trends over the past decade varied by age, sex, and pathogen, adolescent girls and women—especially those 15–24 years old—continue to be disproportionately impacted by both infections and bear the greatest burden of chlamydial infections. Moreover, racial/ethnic disparities in gonorrhea and chlamydia diagnosis rates persist, with non-Hispanic Black people experiencing the highest rates (Centers for Disease Control & Prevention, 2021).

In contrast to increases in reported STI diagnoses, analyses of sexual behaviors with opposite-sex partners and partners overall in the USA since 2000 have found trends generally consistent with stable or decreasing STI transmission (Aholou et al., 2017; Chandra et al., 2012; Copen, 2017; Harper et al., 2017; Leichliter et al., 2020; Murray Horwitz et al., 2018; Twenge et al., 2017; Ueda et al., 2020). One recent analysis found that a composite assessment of behaviors associated with STI transmission remained stable or decreased from 2002 through 2011–15 among adolescent, young adult, Hispanic, and Black heterosexual men and women (Leichliter et al., 2020). With respect to testing behaviors, however, national health systems data suggested that chlamydia testing coverage increased among adolescent girls and young women from the mid-2000s to early 2010s (Hsieh et al., 2017; Tao et al., 2018). Additionally, evidence from national laboratory data and a sentinel surveillance program found that trends in chlamydia and gonorrhea positivity from 2010 to 2017 varied by age, sex, and race/ethnicity but did not reflect the increases in reported diagnoses (Diesel et al., 2021, 2021b; Kaufman et al., 2020; Learner et al., 2020; Niles et al., 2021; U.S. Preventive Services Task Force et al., 2021). However, except Ueda et al. (2020), these analyses of sexual behavior did not cover the more recent period from 2015 to 2019 during which gonorrhea in particular has been rising rapidly (Centers for Disease Control & Prevention, 2021), and most are missing behaviors such as frequency of sex or sexual network attributes, defined as the behaviors and partnership-level characteristics that contribute to linkages among people in sexual networks (Doherty et al., 2005; Weiss et al., 2020). Network attributes, for example concurrency and racial/ethnic mixing, influence the size, speed, and populations impacted in STI epidemics (Rao et al., 2021). Despite STI diagnosis rates increasing among 30–44-year-old women and 15–44-year-old males (Centers for Disease Control & Prevention, 2021), studies of STI outcomes have generally focused on women under 30. In addition, the sentinel surveillance program oversamples populations with higher STI incidence, and health systems data may overestimate access to STI care.

It is therefore unclear to what extent changes in opposite-sex sexual behaviors, sexual network attributes, and STI screening behaviors are associated with increasing diagnoses. To better understand these potential drivers of gonorrhea and chlamydial diagnosis trends, we analyzed temporal trends in sexual behaviors with opposite-sex partners and in self-reported STI testing, treatment, and diagnosis using 2008–2019 data from the National Survey of Family Growth (NSFG), a nationally representative survey of adolescents and adults in US households. Given differences in disease burden and trends in diagnosis rates by sex, age, and race/ethnicity (Centers for Disease Control & Prevention, 2021), we conducted analyses stratified by sex and, within sex, by age and race/ethnicity.

## Method

### Subjects

We analyzed publicly available data from the 2008–10, 2011–13, 2013–15, 2015–17, and 2017–19 NSFG. It involves face-to-face interviews with independent samples of males and females ages 15–44 through the 2013–15 survey period and ages 15–49 from 2015–17 onward. Adolescents ages 15–19, non-Hispanic Black, and Hispanic people are oversampled. Interviewers ask about potential respondents’ biological sex (male or female) as part of the screening process. The survey included a computer-assisted personal interview and audio computer-assisted self-interview (ACASI) for a subset of sensitive questions. Respondents ≥ 18 years provide informed consent; respondents ages 15–17 years provide assent after parental permission. The 2008–2010 data were extracted from the 2006–2010 public-use dataset and included interviews conducted July 2008-June 2010; the other four datasets included interviews conducted September-September in indicated years. NSFG procedures were approved by the National Center for Health Statistics Research Ethics Review Board. Our secondary analysis of de-identified, publicly available data is not considered human subjects research. Details regarding survey design and sample are available elsewhere (National Center for Health Statistics, 2020).

### Measures

We analyzed sexual behaviors associated with heterosexual STI transmission; sexual network attributes; and self-reported STI testing, diagnosis, and treatment. For this analysis, sex with an opposite-sex partner included only vaginal sex. Sexual behaviors included the following with analysis populations in parentheses: ever had sex with an opposite-sex partner (all respondents); sex with an opposite-sex partner in the past 12 months (respondents reporting ever having sex with an opposite-sex partner); number of opposite-sex partners in the past 12 months, condom use at last sex with an opposite-sex partner, ≥ 1 sex act with an opposite-sex partner in the past 4 weeks, and number of sex acts with an opposite-sex partner in the past 4 weeks (those reporting sex with an opposite-sex partner in the past 12 months); and proportion of condom-protected sex acts with an opposite-sex partner in the past 4 weeks (those reporting ≥ 1 sex act with an opposite-sex partner in the past 4 weeks). The last outcome was calculated by dividing the number of condom-protected sex acts by total number of sex acts. Respondents reporting more condom-protected than total sex acts with opposite-sex partners in the past 4 weeks were assumed to have used condoms for every act. This assumption affected 33 (0.2%) of female respondents and 0 male respondents with this outcome.

We defined sexual network attributes as behaviors and partnership-level characteristics that contribute to linkages among people in sexual networks (Doherty et al., 2005; Weiss et al., 2020). These included: having more than 1 “current” opposite-sex partner at time of interview (concurrency point prevalence) (Morris et al., 2009); racial/ethnic homophily with “current” opposite-sex partners (Hamilton & Morris, 2015); and either having had sex with other men in the past 12 months (for male respondents) or having a male partner in the past 12 months who had ever had sex with other men (for female respondents) (Oster et al., 2015). Respondents were considered to have more than 1 current opposite-sex partner if they either (1) reported being married to or living with an opposite-sex partner and reported one or more additional partner (among their most recent 3 partners) to be a “current sexual partner” or (2) reported more than 1 non-marital, non-cohabiting partner to be a “current sexual partner.” Racial/ethnic homophily was measured at the partnership level and included up to 3 “current” partnerships in which sex occurred most recently per respondent (i.e., the partners for whom respondents were asked to report race/ethnicity). It represents the percent of all such partnerships in which both partners are of the same race/ethnicity. Because NSFG public-use datasets include race/ethnicity as: Hispanic, non-Hispanic Black (single race), non-Hispanic White (single race), and non-Hispanic Other or multiple race, we limited analyses of racial/ethnic homophily to Hispanic, non-Hispanic Black, and non-Hispanic White respondents for interpretability. (Note that, because all non-Hispanic persons identifying with multiple races are included in the “Other or multiple race” category in the NSFG public-use dataset, all partnerships between non-Hispanic Black or White respondents [reporting only one race] and multiracial partners are identified as non-homophilous in our analysis, even if one of the partner’s reported races is the same as the respondent’s.) For both concurrency and racial/ethnic homophily, we selected measures that directly reflect the structure and connectivity of the sexual network at a given moment in time and, therefore, its epidemic potential (Morris et al., 2009). For sex with other men or with MSM, we chose the measure that was most directly comparable between male and female respondents. Network attributes were analyzed among respondents reporting sex with an opposite-sex partner in the past 12 months.

Self-reported STI outcomes measured in the past 12 months included: testing for chlamydia (female respondents only), testing for any STI, treated for any STI (“been treated or received medication”), chlamydia diagnosis, and gonorrhea diagnosis. Female respondents were asked about chlamydia testing in all NSFG datasets and testing for all other STIs beginning in 2011–2013; these measures were combined from 2011–2013 onward to assess testing for any STI. Respondents were asked about gonorrhea or chlamydia diagnoses only if they reported STI treatment in the past 12 months in 2008–2010, after which these questions were asked of all respondents. Testing outcomes were analyzed among respondents who reported sex with an opposite-sex partner in the past 12 months. Treatment and diagnosis outcomes were analyzed among respondents who reported testing for any STI and sex with an opposite-sex partner in the past 12 months.

Having sex with other men (for males), male partners who have sex with men (females), and STI outcomes were from the ACASI; all other measures were interview-administered.

### Statistical Analyses

We created two combined datasets, one for female respondents and one for male respondents, defined as biological sex per interviewer administered screening question. To maintain a consistent age range for all years, we limited analyses to respondents ages 15–44 years. All analyses, except for racial/ethnic homophily, were weighted to represent the US household population of males and females at the time of the respective surveys and used individual survey weights. Analyses of racial/ethnic homophily, which we measured for each current partnership among respondents’ three most recent opposite-sex partners, were weighted to represent the current partnerships of the US household population of males and females at the time of the respective surveys. For this outcome, we adjusted weights based on rules for augmented fixed choice design surveys because respondents report their total number of partners but can only name a fixed number of partners (Ott et al., 2019). Partnership weights equaled either (a) respondent’s individual weights if they reported ≤ 3 total current partnerships (i.e., the fixed choice limit, or number of partners the survey asks about), or (b) respondent’s individual weights times their total number of current partners divided by 3.

We evaluated temporal trends using survey-weighted linear or logistic regression. Regression models treated survey year, defined as the mid-point of data collection (i.e., the date the sample was weighted to represent), as a linear independent variable. Resulting β coefficients from linear regression and odds ratios (OR) represent per-year changes in each outcome from 2009–2018. For outcomes reported as means, we also calculated the overall relative change comparing 2008–2010 vs. 2017–2019. Analyses were conducted separately for male and female respondents overall and stratified by age (15–19, 20–29, 30–44 years) and race/ethnicity (Hispanic [any race], non-Hispanic Black [single race], non-Hispanic White [single race]; the latter hereafter referred to as “Black” and “White”). *p*-values < 0.05 were considered statistically significant. Analyses were conducted using survey analysis procedures in SAS® OnDemand for Academics.

## Results

Across all NSFG survey periods in the study, 28,027 female and 24,605 male respondents ages 15–44 were included in this analysis (Supplemental Tables 1 and 2). Demographic characteristics are presented for female and male respondents, respectively, in Supplemental Tables 3 and 4.

### Sexual Behaviors and Network Attributes

#### Female Respondents

**Fig. 1:**
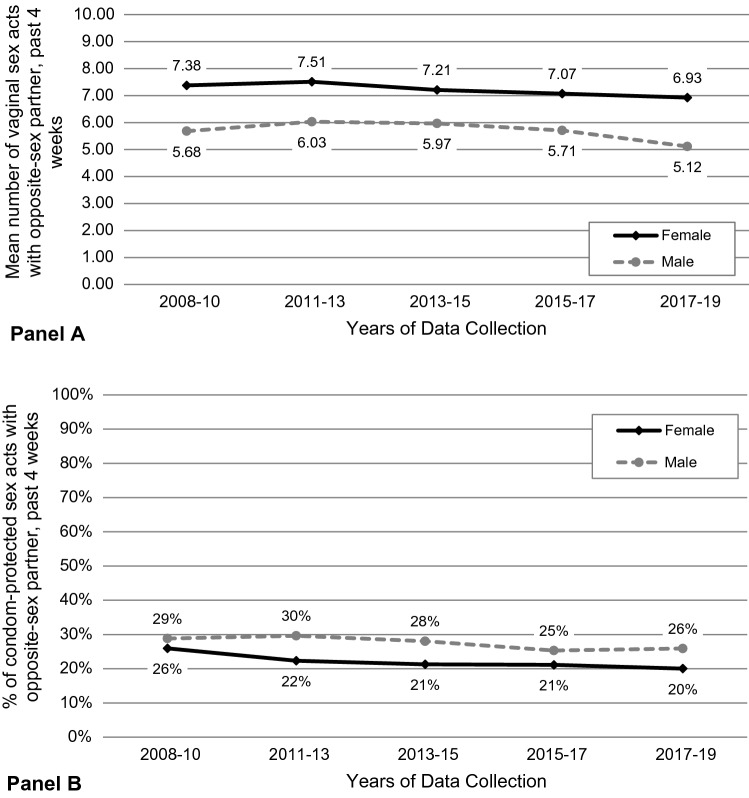
Temporal trends in vaginal sex acts with opposite-sex partners in the past 4 weeks in the National Survey of Family Growth, stratified by respondent sex Panel **A**: Number of vaginal sex acts Panel **B**: Percent of condom-protected vaginal sex acts

Among all female respondents ages 15–44 years, there were significant declines over time in the percent who reported condom use at last sex with a male partner [27.1% in 2008–10 to 24.0% in 2017–19; OR = 0.981, 95% confidence interval (CI) = 0.963–0.999)], mean number of sex acts with male partner(s) in the past 4 weeks (7.38 to 6.39, a relative overall decline of 13%; β = −0.058, 95%CI = −0.109, −0.007), proportion of condom-protected sex acts with male partner(s) in the past 4 weeks (0.259 to 0.200, a relative overall decline of 23%; β = −0.008, 95%CI = −-0.011, −0.004), and proportion of current male partners who were of the same race/ethnicity as the respondent (87.2% to 83.4%; OR = 0.974, 95%CI = 0.952–0.997) (Tables 1 and 3, Fig. 1).

**Table 1:** Sexual behaviors and STI testing, treatment, and diagnosis among female respondents in the 2008–10 to 2017–19 survey periods of the National Survey of Family Growth

	% or Mean (95% Confidence Interval)				
	2008–10	2011–13	2013–15	2015–17	2017–19
Total respondents (unweighted n)	6428	5601	5699	4886	5413
*Sexual behaviors*					
Ever had vaginal sex with male sex partner	87.1%	87.7%	87.3%	86.5%	86.2%
	(85.6–88.6%)	(86.0–89.3%)	(86.1–88.5%)	(84.7–88.3%)	(84.8–87.5%)
Vaginal sex with male sex partner(s) in past 12 months	91.4%	89.7%	90.0%	91.5%	89.3%
	(90.4–92.4%)	(88.4–91.4%)	(88.7–91.4%)	(90.5–92.6%)	(87.8–90.8%)
Number of male sex partners in past 12 months	1.29	1.25	1.28	1.28	1.25
	(1.22–1.36)	(1.20–1.29)	(1.25–1.31)	(1.25–1.31)	(1.21–1.28)
Condom use at last vaginal sex	27.1%	25.5%	24.6%	23.9%	24.0%
	(24.7–29.7%)	(23.6–27.4%)	(22.5–26.6%)	(21.3–26.5%)	(21.7–26.3%)
≥ 1 vaginal sex act in past 4 weeks	82.6%	81.8%	82.6%	82.8%	83.5%
	(80.9–84.2%)	(79.8–83.7%)	(80.9–84.3%)	(80.8–84.8%)	(81.7–85.2%)
Number of vaginal sex acts in past 4 weeks	7.38	7.51	7.21	7.07	6.93
	(7.04–7.71)	(7.15–7.88)	(6.88–7.53)	(6.64–7.50)	(6.57–7.28)
Proportion of condom-protected vaginal sex acts in past 4 weeks	0.259	0.223	0.213	0.211	0.200
	(0.234–0.285)	(0.205–0.242)	(0.192–0.234)	(0.185–0.237)	(0.179–0.221)
Vaginal, oral, or anal sex with a man who has sex with men	2.0%	1.9%	2.0%	2.0%	2.8%
	(1.4–2.5%)	(1.3–2.5%)	(1.5–2.5%)	(1.3–2.7%)	(2.0–3.6%)
Racial/ethnic homophily among up to 3 current partners with whom vaginal sex occurred most recently*	87.2%	83.6%	83.7%	84.2%	83.4%
	(85.4–88.9%)	(81.5–85.8%)	(81.5–85.8%)	(81.5–86.9%)	(81.1–85.8%)
Concurrency (≥ 2 current partners at time of survey)	1.0%	1.2%	0.8%	1.4%	1.3%
	(0.7–1.3%)	(0.8–1.6%)	(0.6–1.0%)	(0.8–1.9%)	(0.8–1.7%)
*STI Testing, Treatment, and Diagnosis*					
Chlamydia testing in past 12 months	26.4%	27.5%	30.6%	32.0%	30.0%
	(24.3–28.4%)	(25.0–30.1%)	(28.4–32.8%)	(30.0–34.0%)	(27.7–32.3%)
STI testing in past 12 months^	NA	32.3%	36.0%	37.8%	35.6%
		(29.7–34.9%)	(33.5–38.4%)	(35.8–39.8%)	(33.1–38.0%)
STI treatment in past 12 months^	NA	13.4%	12.9%	11.3%	13.6%
		(10.7–16.2%)	(10.8–15.0%)	(8.5–14.1%)	(11.2–16.1%)
Gonorrhea diagnosis in past 12 months^	NA	2.1%	1.7%	2.0%	3.1%
		(1.3–2.9%)	(1.1–2.2%)	(1.1–2.8%)	(2.1–4.0%)
Chlamydia diagnosis in past 12 months^	NA	4.6%	4.4%	5.7%	4.8%
		(3.3–5.8%)	(3.2–5.6%)	(3.4–8.0%)	(3.4–6.2%)

NA = not applicable/available

All estimates account for survey weights

^*^Measured among Hispanic, Non-Hispanic Black, and Non-Hispanic White respondents only

^Analysis includes 2011–13 through 2017–19 survey periods only. In the 2008–10 period, female respondents were not asked about STI testing other than chlamydia and were not asked about gonorrhea or chlamydia diagnoses unless they reported STI treatment in the past 12 months

Stratified by age, the declines in behaviors and racial/ethnic homophily observed among females overall were significant only among those ages 20–29 (Table 3). This age group also reported significant declines in the percent who had ever had sex with a male partner, sex with a male partner in the past 12 months, and sex in the past 12 months with a man who had ever had sex with men. No significant temporal trends were observed in sexual behaviors or network determinants among females ages 15–19 or 30–44. Some associations were consistent across time: reporting ever having sex and ≥ 1 sex act in the past 4 weeks increased as age increased, number of sex acts in the past 4 weeks was highest among 20–29-year-olds, and reported number of partners in the past 12 months and condom use declined with increasing age (Supplemental Table 5).

Similarly, racial/ethnic differences in sexual behaviors and network attributes among females persisted over time (Table 3, Supplemental Table 7). The percent reporting ever having had sex with a male partner decreased significantly over time among Hispanic females only. Among Black females, condom use at last sex, number of sex acts in the past 4 weeks, proportion of condom-protected sex acts in the past 4 weeks, and racial/ethnic homophily with current male partners decreased significantly. White females also reported significant declines in the proportion of condom-protected sex acts in the past 4 weeks. Of note, Black and White females reported higher numbers of male partners in the past 12 months than Hispanic females, and condom use was highest among Black females followed by Hispanic then White females using both measures.

#### Male Respondents

Among all male respondents ages 15–44, there were significant decreases from 2008–10 to 2017–19 in mean number of female partners in the past 12 months (1.45 to 1.35, a relative overall decline of 7%; β = -0.012, 95%CI = -0.018, -0.005), percent reporting condom use at last sex with a female partner (34.6% to 31.6%; OR = 0.981, 95%CI = 0.966–0.996), mean number of sex acts with female partner(s) in the past 4 weeks (5.68 to 5.12 acts, a relative overall decline of 10%; β = -0.059, 95%CI = -0.116, -0.001), proportion of condom-protected sex acts with female partner(s) in the past 4 weeks (0.288 to 0.259, a relative overall decline of 10%; β = -0.004, 95%CI = -0.004, -0.001), percent of current female partners who were of the same race/ethnicity as the respondent (86.1% to 80.8%; OR = 0.974, 95%CI = 0.952–0.997), and percent reporting ≥ 2 current partnerships with female partners (2.8% to 1.5%; OR = 0.943, 95%CI = 0.902–0.985) (Table 2, Fig. 1). Among males who had a female sex partner in the past 12 months, the percent who reported ever having had oral or anal sex with another male increased from 3.5% in 2008–10 to 5.0% in 2017–19 (OR = 1.037, 95%CI = 1.003–1.072). (Table 3)

**Table 2:** Sexual behaviors and STI testing, treatment, and diagnosis among male respondents in the 2008–10 to 2017–19 survey periods of the National Survey of Family Growth

	% or Mean (95% Confidence Interval)				
	2008–10	2011–13	2013–15	2015–17	2017–19
Total respondents (unweighted n)	5538	4815	4506	3998	4622
*Sexual behaviors*					
Ever had vaginal sex with female sex partner	85.6%	86.6%	86.5%	84.7%	84.3%
	(84.7–86.5%)	(85.2–87.9%)	(85.3–87.7%)	(83.2–86.2%)	(82.6–86.0%)
Vaginal sex with female sex partner(s) in past 12 months	91.0%	90.7%	91.0%	90.5%	90.3%
	(89.3–92.2%)	(89.6–91.8%)	(89.7–92.2%)	(89.2–91.8%)	(88.7–91.9%)
Number of female sex partners in past 12 months	1.45	1.46	1.42	1.40	1.35
	(1.41–1.49)	(1.41–1.51)	(1.36–1.47)	(1.35–1.44)	(1.31–1.39)
Condom use at last vaginal sex	34.6%	36.6%	34.0%	32.5%	31.6%
	(32.5–36.6%)	(34.6–38.6%)	(31.4–36.5%)	(29.5–35.4%)	(28.9–34.2%)
≥ 1 vaginal sex act in past 4 weeks	81.8%	81.1%	81.0%	81.0%	78.5%
	(80.0–83.6%)	(79.5–82.7%)	(79.4–82.6%)	(79.0–83.1%)	(75.8–81.1%)
Number of vaginal sex acts in past 4 weeks	5.68	6.03	5.97	5.71	5.12
	(5.24–6.12)	(5.71–6.35)	(5.65–6.29)	(5.36–6.06)	(4.75–5.48)
Proportion of condom-protected vaginal sex acts in past 4 weeks	0.288	0.296	0.280	0.253	0.259
	0.265–0.311)	(0.270–0.322)	(0.251–0.310)	(0.230–0.276)	(0.234–0.284)
Ever had oral or anal sex with another man	3.5%	4.2%	3.5%	4.3%	5.0%
	(2.7–4.3%)	(3.2–5.2%)	(2.7–4.3%)	(3.1–5.5%)	(4.1–5.9%)
Racial/ethnic homophily among up to 3 current partners with whom vaginal sex occurred most recently^*^	86.1%	83.0%	83.0%	79.5%	80.8%
	(84.2–88.1%)	(80.4–85.5%)	(80.2–85.7%)	(76.4–82.6%)	(78.4–83.1%)
Concurrency (≥ 2 current partners at time of survey)	2.8%	2.4%	2.6%	2.0%	1.5%
	(2.0–3.6%)	(1.9–3.0%)	(1.8–3.3%)	(1.2–2.9%)	(1.0–2.0%)
*STI Testing, Treatment, and Diagnosis*					
STI testing in past 12 months	16.7%	17.3%	16.3%	17.0%	15.0%
	(14.7–18.8%)	(15.5–19.0%)	(14.5–18.0%)	(14.9–19.1%)	(13.4–16.7%)
STI treatment in past 12 months	15.7%	11.6%	13.6%	13.7%	10.6%
	(12.0–19.3%)	(8.6–14.7%)	(10.4–16.8%)	(9.1–18.4%)	(8.0–13.2%)
Gonorrhea diagnosis in past 12 months^	NA	2.3%	2.2%	3.0%	2.1%
		(1.3–3.3%)	(1.1–3.4%)	(1.3–4.7%)	(1.0–3.3%)
Chlamydia diagnosis in past 12 months^	NA	3.8%	5.4	6.0%	3.3%
		(2.7–5.0%)	(3.2–7.6%)	(2.6–9.3%)	(1.5–5.1%)

NA = not applicable/available

All estimates account for survey weights

^*^Measured among Hispanic, Non-Hispanic Black, and Non-Hispanic White respondents only

^Analysis includes 2011–13 through 2017–19 survey periods only. In the 2008–10 survey period, male respondents were not asked about gonorrhea or chlamydia diagnoses unless they reported STI treatment in the past 12 months

**Table 3:** Estimates of the annual change in sexual behaviors and STI testing, treatment, and diagnosis among female respondents in the 2008–10 through 2017–19 survey periods of the National Survey of Family Growth

	Odds ratios or β (95%Confidence Interval)						
	Overall	15–19	20–29	30–44	Hispanic	NH Black	NH White
*Sexual behaviors*							
Ever had vaginal sex with male sex partner	0.989	0.981	**0.953**	0.992	**0.961**	1.010	0.986
	(0.970, 1.008)	(0.955, 1.007)	**(0.918, 0.990)**	(0.932, 1.056)	**(0.935, 0.987)**	(0.972, 1.049)	(0.959, 1.014)
Vaginal sex with male sex partner(s) in past 12 months	0.987	0.989	**0.958**	1.001	1.011	0.992	0.980
	(0.966, 1.007)	(0.930, 1.052)	**(0.926, 0.992)**	(0.976, 1.026)	(0.974, 1.049)	(0.953, 1.032)	(0.953, 1.009)
Number of male sex partners in past 12 months*	−0.003	−0.003	−0.006	-0.007	0.000	−0.007	−0.002
	(−0.011, 0.005)	(−0.031, 0.025)	(−0.005, 0.016)	(−0.019, 0.010)	(−0.008, 0.008)	(−0.019, 0.005)	(−0.014, 0.011)
Condom use at last vaginal sex	**0.981**	0.977	**0.967**	1.001	0.990	**0.968**	0.978
	**(0.963, 0.999)**	(0.934, 1.023)	**(0.943, 0.992)**	(0.975, 1.026)	(0.960, 1.020)	**(0.941, 0.996)**	(0.954, 1.002)
≥ 1 vaginal sex act in past 4 weeks	1.008	0.997	1.004	1.009	1.016	1.021	1.009
	(0.991, 1.026)	(0.958, 1.037)	(0.976, 1.033)	(0.986, 1.032)	(0.986, 1.048)	(0.989, 1.054)	(0.984, 1.034)
Number of vaginal sex acts in past 4 weeks*	**−0.058**	−0.128	**−0.111**	−0.014	−0.045	**−0.117**	−0.059
	**(−0.109, −0.007)**	(−0.262, 0.010)	**(−0.200, −0.020)**	(−0.085, −0.060)	(−0.122, 0.033)	**(−0.225, −0.009)**	(−0.127, 0.010)
Proportion of condom-protected vaginal sex acts in past 4 weeks *	**−0.008**	−0.003	**−0.011**	−0.002	−0.005	**−0.008**	**−0.005**
	**(−0.011, −0.004)**	(−0.016, 0.010)	**(−0.016, −0.006)**	(−0.005, 0.002)	(−0.012, 0.002)	**(−0.015, 0.000)**	**(−0.009, −0.002)**
Vaginal, oral, or anal sex with a man who has sex with men	1.039	1.009	**1.080**	1.013	1.029	1.093	1.051
	(0.992, 1.089)	(0.886, 1.149)	**(1.004, 1.163)**	(0.943, 1.088)	(0.934, 1.134)	(0.993, 1.205)	(0.977, 1.130)
Racial/ethnic homophily among up to 3 current partners with whom vaginal sex occurred most recently^	**0.974**	0.952	**0.965**	0.985	0.999	0.952	1.093
	**(0.952, 0.997)**	(0.891, 1.017)	**(0.933, 0.998)**	(0.953,1.017)	(0.958, 1.041)	(0.892, 1.017)	(0.993, 1.203)
Concurrency (≥ 2 current partners at time of survey)	1.027	0.915	1.035	1.043	1.093	**0.913**	1.063
	(0.976, 1.080)	(0.760, 1.102)	(0.943, 1.135)	(0.972, 1.119)	(0.993, 1.203)	**(0.852, 0.979)**	(0.972, 1.162)
*STI Testing, Treatment, and Diagnosis*							
Chlamydia testing in past 12 months	**1.027**	0.962	1.021	**1.055**	1.032	**1.038**	1.019
	**(1.011, 1.042)**	(0.923, 1.004)	(0.999, 1.044)	**(1.034, 1.077)**	(1.000, 1.064)	**(1.008, 1.069)**	(0.997, 1.041)
STI testing in past 12 months†	**1.026**	0.994	1.018	**1.045**	1.005	1.017	1.029
	**(1.001, 1.051)**	(0.927, 1.067)	(0.979, 1.057)	**(1.013, 1.078)**	(0.958, 1.055)	(0.976, 1.068)	(0.997, 1.062)
STI treatment in past 12 months†	0.996	1.021	0.989	1.013	0.998	0.999	1.002
	(0.945, 1.048)	(0.914, 1.141)	(0.918, 1.064)	(0.931, 1.101)	(0.899, 1.109)	(0.922, 1.083)	(0.907, 1.108)
Gonorrhea diagnosis in past 12 months†	1.082	**1.411**	1.021	1.088	1.009	1.074	1.128
	(0.986, 1.186)	**(1.131, 1.761)**	(0.895, 1.165)	(0.944, 1.254)	(0.808, 1.261)	(0.919, 1.255)	(0.978, 1.301)
Chlamydia diagnosis in past 12 months†	1.021	1.059	1.038	0.993	0.926	1.038	1.073
	(0.957–1.091)	(0.926, 1.211)	(0.953, 1.131)	(0.864, 1.141)	(0.809, 1.061)	(0.938, 1.150)	(0.943, 1.221)

Bold indicates significance at < 0.05 level

^*^Estimates derived from linear regression (β). All others from logistic regression (odds ratios). All models accounted for survey weights. All estimates represent per-year changes in each outcome from 2009–2018

^Measured among Hispanic, Non-Hispanic Black, and Non-Hispanic White respondents only

^†^Analysis includes 2011–13 through 2017–19 survey periods only. In the 2008–10 survey period, female respondents were not asked about STI testing other than chlamydia and were not asked about gonorrhea or chlamydia diagnoses unless they reported STI treatment in the past 12 months

Stratified by age, the mean number of female partners in the past 12 months and number of sex acts in the past 4 weeks reported by males ages 15–19 and 20–29; proportion of condom-protected sex acts in the past 4 weeks reported by men ages 20–29; and percent of men ages 30–44 reporting ever having had sex with female partner(s) all declined over time (Table 4). In addition, reporting ever having had oral or anal sex with another male increased significantly among 15–19 and 30–44-year-olds; racial/ethnic homophily with current female partners decreased significantly among 20–29 and 30–44-year-olds; and concurrency decreased significantly among 20–29-year-olds. Similar to females, reporting ever having sex and ≥ 1 sex act in the past 4 weeks increased with increasing age, reported number of partners in the past 12 months and condom use declined with increasing age, and number of sex acts in the past 4 weeks was highest among 20–29-year-olds in all years (Supplemental Table 5).

**Table 4:** Estimates of the annual change in sexual behaviors and STI testing, treatment, and diagnosis among male respondents in the 2008–10 through 2017–19 survey periods of the National Survey of Family Growth

	Odds ratios or β (95% Confidence Interval)						
	Overall	15–19	20–29	30–44	Hispanic	NH Black	NH White
*Sexual behaviors*							
Ever had vaginal sex with female sex partner	0.985	0.978	0.970	**0.937**	0.973	1.000	0.989
	(0.970, 1.001)	(0.954, 1.003)	(0.925, 1.017)	**(0.881, 0.997)**	(0.944, 1.002)	(0.962, 1.040)	(0.968, 1.012)
Vaginal sex with female sex partner(s) in past 12 months	0.991	0.951	0.983	1.005	0.979	**0.944**	1.008
	(0.969, 1.014)	(0.902, 1.003)	(0.951, 1.016)	(0.975, 1.035)	(0.931, 1.029)	**(0.900, 0.990)**	(0.977, 1.039)
Number of female sex partners in past 12 months*	**−0.012**	**−0.035**	**−0.014**	−0.004	−0.010	−0.017	**−0.011**
	**(−0.018, −0.005)**	**(−0.057, −0.013)**	**(−0.027, −0.002)**	(−0.011, 0.002)	(−0.024, 0.003)	(−0.042, 0.007)	**(−0.019, −0.004)**
Condom use at last vaginal sex	**0.981**	0.971	0.978	0.991	0.975	**0.965**	**0.978**
	**(0.966, 0.996)**	(0.930, 1.013)	(0.955, 1.001)	(0.971, 1.013)	(0.949, 1.003)	**(0.938, 0.992)**	**(0.959, 0.998)**
≥ 1 vaginal sex act in past 4 weeks	0.981	0.988	0.969	0.983	0.981	0.968	0.984
	(0.961, 1.001)	(0.949, 1.029)	(0.938, 1.000)	(0.954, 1.014)	(0.938, 1.025)	(0.927, 1.010)	(0.959, 1.011)
Number of vaginal sex acts in past 4 weeks*	**−0.059**	**−0.127**	**−0.119**	−0.018	−0.110	−0.072	−0.035
	**(−0.116, −0.001)**	**(−0.240, −0.014)**	**(−0.209, −0.028)**	(−0.093, 0.056)	(−0.276, 0.055)	(−0.183, 0.039)	(−0.093, 0.003)
Proportion of condom−protected vaginal sex acts in past 4 weeks*	**−0.004**	−0.008	**−0.008**	−0.001	−0.004	**−0.008**	**−0.006**
	**(−0.008, −0.001)**	(−0.019, 0.004)	**(−0.013, −0.002)**	(−0.005, 0.003)	(−0.011, 0.003)	**(−0.015, −0.001)**	**(−0.011, −0.002)**
Ever had oral or anal sex with another man	**1.037**	**1.020**	1.014	**1.052**	1.071	0.967	1.034
	**(1.003, 1.072)**	**(0.907, 1.148)**	(0.950, 1.082)	**(1.008, 1.097)**	(0.990, 1.158)	(0.896, 1.044)	(0.994, 1.076)
Racial/ethnic homophily among up to 3 current partners with whom vaginal sex occurred most recently^	**0.954**	0.948	**0.934**	**0.965**	0.971	**0.895**	**0.954**
	**(0.933, 0.977)**	(0.899, 1.011)	**(0.905, 0.965)**	**(0.937, 0.994)**	(0.935, 1.011)	**(0.895, 0.978)**	**(0.919, 0.992)**
Concurrency (≥ 2 current partners at time of survey)	**0.943**	0.970	**0.932**	0.946	0.980	0.929	**0.925**
	**(0.902, 0.985)**	(0.890, 1.057)	**(0.869, 1.000)**	(0.885, 1.011)	(0.908, 1.058)	(0.861, 1.002)	**(0.867, 0.987)**
*STI Testing, Treatment, and Diagnosis*							
STI testing in past 12 months	0.989	0.959	**0.968**	1.016	0.997	0.974	0.977
	(0.969, 1.009)	(0.918, 1.001)	**(0.937, 1.000)**	(0.989, 1.044)	(0.964, 1.032)	(0.942,1.007)	(0.948, 1.007)
STI treatment in past 12 months	0.989	**0.819**	1.031	1.021	0.979	0.973	0.973
	(0.929, 1.053)	**(0.694, 0.966)**	(0.951, 1.117)	(0.914, 1.142)	(0.836, 1.146)	(0.875, 1.081)	(0.875, 1.081)
Gonorrhea diagnosis in past 12 months†	0.948	1.052	0.985	1.033	0.929	0.990	1.009
	(0.868, 1.035)	(0.821, 1.347)	(0.839, 1.156)	(0.862, 1.238)	(0.679, 1.272)	(0.854, 1.148)	(0.826, 1.232)
Chlamydia diagnosis in past 12 months†	0.994	1.032	1.035	0.940	1.041	0.988	1.008
	(0.911, 1.085)	(0.873, 1.221)	(0.921, 1.164)	(0.801, 1.103)	(0.793, 1.366)	(0.810, 1.146)	(0.868, 1.172)

Bold indicates significance at < 0.05 level

^*^Estimates derived from linear regression (β). All others from logistic regression (odds ratios). All models accounted for survey weights. All estimates represent per-year changes in each outcome from 2009 to 2018

^Measured among Hispanic, Non-Hispanic Black, and Non-Hispanic White respondents only

^†^Analysis includes 2011–13 through 2017–19 survey periods only. In the 2008–10 survey period, respondents were not asked about gonorrhea or chlamydia diagnoses unless they reported STI treatment in the past 12 months

Stratified by race/ethnicity, the proportion reporting sex with a female partner in the past 12 months and ≥ 2 current female partners decreased significantly over time among Black males only; mean number of female partners in the past 12 months decreased significantly among White males only; and condom use at last sex and during sex in the past 4 weeks and racial/ethnic homophily with current female partners decreased among both Black and White males (Table 4). No significant temporal trends were observed among Hispanic males.

### Self-Reported Sexually Transmitted Infection Outcomes

#### Female Respondents

Among all female respondents ages 15–44, the percent reporting testing for chlamydia (26.4% in 2008–10 to 30.0% in 2017–19; OR = 1.027, 95%CI = 1.011–1.042) and any STI (32.3% in 2011–13 to 35.6% in 2017–19; OR = 1.026, 95%CI = 1.001–1.051) increased significantly over time (Table 1). These increases were significant among females ages 30–44 but not younger age groups (Table 3), who were more likely to report testing across all years (Supplemental Table 5). Among those screened for STIs in the past 12 months, no significant temporal trends were observed in STI treatment, gonorrhea diagnosis, or chlamydia diagnosis overall, by age, or by race/ethnicity, except for an increase in gonorrhea diagnoses among females ages 15–19 (Table 3). In all years, Black females were more likely to report testing than either Hispanic or White females, and Black and Hispanic females were more likely to report gonorrhea and chlamydia diagnoses than White females (Supplemental Table 7).

#### Male Respondents

There were no significant changes in self-reported STI testing, treatment, or diagnosis over time among all male respondents ages 15–44 or within racial/ethnic groups (*p* > 0.05 for all, Tables 2 and 4). However, non-Hispanic Black males were more likely than Hispanic and White males to report each STI outcome in all years. Reported STI testing in the past 12 months decreased from 2008–10 to 2017–19 among 15–19 (OR = 0.959; 95%CI = 0.937–1.001) and 20–29-year-olds (0.968; 0.937–1.000) but was stable among 30–44-year-olds (1.016; 0.989–1.044) [Table 4]. Among those tested in the past 12 months, STI treatment decreased significantly among 15–19-year-old males (OR = 0.819; 95%CI = 0.694–0.966) but was stable in older age groups. However, younger males were more likely to report testing, treatment, and diagnoses in general.

Estimates for stratified outcomes are presented in Supplemental Tables 5 (females by age), 6 (males by age), 7 (females by race/ethnicity), and 8 (males by race/ethnicity).

## Discussion

From 2008–10 through 2017–19, we observed a complex set of changes in sexual behaviors, sexual network attributes, and STI outcomes among US females and males ages 15–44 with opposite-sex partners. Overall, reports of sexual activity, condom use during vaginal sex, racial/ethnic homophily, and concurrency decreased, while the proportion of males reporting sex with other males increased. These changes were most apparent among 20–29-year-old females and males, Black females and males, and White males. Racial/ethnic differences persisted over time, most notably that condom use was highest among Black respondents followed by Hispanic then White respondents. At the same time, STI testing in the past year increased among females ages 30–44 and Black females but decreased among males ages 20–29.

Previous analyses of NSFG as recently as 2011–2015 found behavioral and network trends consistent with stable or decreasing STI transmission, including no changes in numbers of opposite-sex partners (Chandra et al., 2012; Harper et al., 2017; Murray Horwitz et al., 2018) or concurrent or non-monogamous partnerships (Aholou et al., 2017) reported by both male and female respondents; decreases in exchanging sex for money or drugs (Chandra et al., 2012); stable or increasing condom use at last sex among female and male respondents, respectively (Aholou et al., 2017; Copen, 2017); and stable or decreasing STI risk among several groups with high STI incidence (Leichliter et al., 2020). Similarly, two analyses of sexual frequency with partners of any sex and total partner number in the General Social Survey, another nationally representative survey, found trends consistent with stable or decreasing sexual activity (Twenge et al., 2017; Ueda et al., 2020). By contrast, our findings include changes that could lead to increased STI transmission (decreases in condom use), decreased transmission (decreases in number of sex acts in the past 4 weeks among male and females, numbers of opposite-sex partners in the past year and concurrency among males), and with less clear impacts on transmission (increased racial/ethnic mixing). Changes in racial/ethnic homophily alter the network structure and have the potential to increase or decrease the rate of transmission as well as the risk of exposure at the individual level. Transmission within sexual networks is a dynamic process, and the impact of changing mixing patterns on transmission at the population-level is challenging to ascertain, particularly in the shorter term. Differences between our findings and previous studies may, in part, be due to actual changes in behaviors and network attributes represented by inclusion of later NSFG survey periods. However, differences in our initial reference period and analytic strategies may also have contributed. Previous analyses of NSFG data examined trends beginning in 2002 or 2006–8 rather than 2008–10 and treated survey period as a nominal variable to compare individual periods rather than a linear variable analyzing overall trends (Aholou et al., 2017; Chandra et al., 2012; Copen, 2017; Harper et al., 2017; Leichliter et al., 2020; Murray Horwitz et al., 2018).

Overall changes in sexual behaviors and network attributes over time have potential to obscure important subpopulation-specific differences and changes that contribute to the age-specific trends in reported diagnoses and persistent racial/ethnic inequities in STIs (Centers for Disease Control & Prevention, 2021). Our observed changes in sexual behaviors and network attributes were mostly significant among respondents ages 20–29, corresponding to the age groups that experienced the greatest increases in reported gonorrhea and chlamydia diagnosis rates during the study period (Centers for Disease Control & Prevention, 2021). These changes were also significant primarily among Black and White males, despite all racial/ethnic groups experiencing relatively similar increases in diagnosis rates over time (Centers for Disease Control & Prevention, 2021). Although not a focus of our analyses, these changes may also differ by subpopulations defined by STI risk or marital status. For example, a recent analysis of NSFG data from 2002 through 2017 found that condom use declined among unmarried, non-cohabiting males ages 15–29 who have sex with females who reported STI risk factors but increased among those without such risk factors (Copen et al., 2020). Together, these highlight the importance of subpopulation-specific trends for understanding STI transmission and identifying groups to prioritize for behavior change interventions.

The U.S. Preventive Services Task Force recommends chlamydia and gonorrhea screening for all sexually active females under 25 and among women 25 years and older who are at increased risk, but does not currently recommend such screening for men (U.S. Preventive Services Task Force et al., 2021), though CDC does recommend at least annual screening for men who have sex with men (Workowski et al., 2021). In our study, only one-third of females ages 15–19 and two in five women ages 20–29 reported testing for any STI in the past year, and these numbers remained stable over the past decade, indicating the need to increase efforts to achieve universal screening. Encouragingly, Black females—the group with the highest rates of reported STI diagnoses and highest baseline testing rate—and women ages 30–44 reported increased testing over this period. However, women ages 30–44 remained much less likely to have tested than their younger counterparts. Previous analyses of national medical claims data suggested that chlamydia testing coverage among sexually active females ages 15–25 increased substantially from the mid-2000s to early 2010s. However, the increase in chlamydia testing slowed after 2009 (Hsieh et al., 2017; Tao et al., 2018), the period included in our analyses.

In addition, despite substantial increases in reported chlamydia and gonorrhea diagnoses in the USA over this period (Centers for Disease Control & Prevention, 2021), we found no significant trends in the percent of those tested who reported gonorrhea or chlamydia diagnoses overall or within subgroups, except an increase in the percent diagnosed with gonorrhea among 15–19-year-old females. By comparison, national laboratory testing data indicated that gonorrhea and chlamydia test positivity decreased from 2010 to 2017 among 12–17-year-old females but increased among women ages 18–30 and pregnant persons (Kaufman et al., 2020; Niles et al., 2021). Among National Job Training Program participants ages 16–24, gonorrhea prevalence increased among females and decreased among males from 2011 to 2017 (Learner et al., 2020), and chlamydia prevalence increased among 20–24-year-old White women, decreased among 20–24-year-old Black men from 2010 to 2017, and remained stable among other groups (Diesel et al., 2021a, 2021b). Each dataset has important limitations that may contribute to the differences in findings. Notably, laboratory testing data are of unknown representativeness, the National Job Training Program oversamples populations with high STI incidence, and NSFG is nationally representative but relies on self-report. Additionally, except chlamydia testing among women, STI testing and treatment outcomes in NSFG are not pathogen-specific. Therefore, they may not reflect gonorrhea or chlamydia testing and treatment specifically, and gonorrhea and chlamydia positivity may be underestimated insofar as respondents report testing for other STIs.

Our study has additional limitations. First, all outcomes were self-reported—many in face-to-face interactions—and therefore sensitive to recall and social desirability bias. Second, in the absence of a question about gender identity in NSFG, our analyses were limited to respondents’ biological sex, and our analyses focused on vaginal sex, despite the potential contributions of oral and anal sex to STI transmission (Ballini et al., 2012; Khosropour et al., 2021; Stewart et al., 2021). Third, we were unable to report outcomes and trends specific to Asian, Native Hawaiian/Pacific Islander, Native American/Alaska Native, or multiracial respondents due to smaller sample sizes for these groups. In addition, due to limited availability of partner race/ethnicity data, we analyzed racial/ethnic homophily among up to three current partnerships only, which may not be representative of all partnerships, particularly those of short duration. We were also unable to examine trends in age mixing because partner age was not available in the public dataset after 2015 or in mixing by sexual activity, which was not assessed, despite their importance to STI transmission (Easterly et al., 2018; Newman, 2002; Rao et al., 2021). More generally, the way partners connect has been changing rapidly (Anderson et al., 2020; Rosenfeld et al., 2019), which could have significant implications for the sexual network’s structure and epidemic potential, and these data were collected prior to the COVID-19 pandemic, which impacted sexual behaviors, networks, and STI screening (Masoudi et al., 2022; Mourikis et al., 2022). Finally, we made multiple comparisons and may have observed some significant differences due to chance.

It is unclear how the relatively small and diverging changes in sexual behavior and network attributes coupled with age- and sex-specific changes in testing we observed may have affected STI transmission and diagnosis over the past decade. However, it seems unlikely that these alone can explain the large increases in reported gonorrhea and chlamydia diagnoses. Research, including transmission modeling, is needed to determine how the observed changes in sexual behavior, network attributes, and STI testing intersect to explain the increases in reported cases and identify appropriate targets for future intervention to reduce STI transmission.

## Supplementary Information

Below is the link to the electronic supplementary material.

Supplementary file1 (DOCX 126 kb)

## Declarations

### Availability of Data and Materials

The study utilized public use data files from the National Survey of Family Growth, which are available at https://www.cdc.gov/nchs/nsfg/index.htm.

### Code Availability

Analyses were conducted in SAS® OnDemand for Academics. Code is available upon request.
