# Supplementary material for: Changes in Sexual Behaviors with Opposite-Sex Partners and Sexually Transmitted Infection Outcomes Among Females and Males Ages 15–44 Years in the USA: National Survey of Family Growth, 2008–2019
Source: Arch Sex Behav. 2022 Dec 6;52(2):809–21. doi: 10.1007/s10508-022-02485-3 (PMC9735137; doi:10.1007/s10508-022-02485-3)
Supplement: Supplementary file 1 — Supplementary file1 (DOCX 126 kb) [file 10508_2022_2485_MOESM1_ESM.docx]

**Changes in Sexual Behaviors with Opposite Sex Partners and STI Outcomes among Females and Males Ages 15-44 in the United States: National Survey of Family Growth, 2008-2019**

***Supplemental Tables***

**SUPPLEMENTAL TABLES**

| **Supplemental Table 1. Unweighted Sample Sizes for Key Denominators among Female Respondents in the 2008-10 to 2017-19 survey periods of the National Survey of Family Growth** | | | | | | |
| --- | --- | --- | --- | --- | --- | --- |
| **Outcome** | **Group** | **2008-10** | **2011-13** | **2013-15** | **2015-17** | **2017-19** |
| **Total respondents** | Overall | 6428 | 5601 | 5699 | 4886 | 5413 |
|  | 15-19 yrs | 1199 | 1037 | 1010 | 924 | 970 |
|  | 20-29 yrs | 2345 | 2030 | 2000 | 1678 | 1828 |
|  | 30-44 yrs | 2884 | 5601 | 2689 | 2284 | 2615 |
|  | Hispanic | 1546 | 1458 | 1394 | 1101 | 1541 |
|  | NH Black | 1264 | 1171 | 1114 | 1100 | 1062 |
|  | NH White | 3119 | 2493 | 2589 | 2208 | 2336 |
|  | Another race/ethnicity* | 499 | 479 | 602 | 477 | 474 |
| **Respondents who reported vaginal sex with a male sex partner in the past 12 months** | Overall | 4966 | 4319 | 4363 | 3701 | 4038 |
|  | 15-19 yrs | 527 | 420 | 384 | 329 | 359 |
|  | 20-29 yrs | 2002 | 1735 | 1653 | 1424 | 1460 |
|  | 30-44 yrs | 2437 | 2164 | 2326 | 1948 | 2219 |
|  | Hispanic | 1182 | 1104 | 1029 | 818 | 1112 |
|  | NH Black | 1003 | 897 | 862 | 836 | 788 |
|  | NH White | 2416 | 1978 | 2038 | 1699 | 1778 |
|  | Another race/ethnicity* | 365 | 340 | 434 | 348 | 360 |
| *Includes non-Hispanic respondents reporting multiple races. | | | | | | |

| **Supplemental Table 2. Unweighted Sample Sizes for Key Denominators among Female Respondents in the 2008-10 to 2017-19 survey periods of the National Survey of Family Growth** | | | | | | |  |
| --- | --- | --- | --- | --- | --- | --- | --- |
| **Outcome** | **Group** | **2008-10** | **2011-13** | **2013-15** | **2015-17** | **2017-19** | |
| **Total respondents** | Overall | 5538 | 4815 | 4506 | 3998 | 4622 | |
|  | 15-19 yrs | 1302 | 1088 | 999 | 886 | 1032 | |
|  | 20-29 yrs | 1884 | 1664 | 1521 | 1336 | 1435 | |
|  | 30-44 yrs | 2352 | 2063 | 1986 | 1776 | 2155 | |
|  | Hispanic | 1341 | 1172 | 1013 | 898 | 1251 | |
|  | NH Black | 992 | 873 | 775 | 754 | 803 | |
|  | NH White | 2718 | 2315 | 2221 | 1928 | 2114 | |
|  | Another race/ethnicity* | 487 | 465 | 497 | 418 | 454 | |
| **Respondents who reported vaginal sex with a male sex partner in the past 12 months** | Overall | 4075 | 3590 | 3305 | 2901 | 3335 | |
|  | 15-19 yrs | 531 | 475 | 392 | 319 | 386 | |
|  | 20-29 yrs | 1521 | 1335 | 1206 | 1036 | 1115 | |
|  | 30-44 yrs | 2023 | 1780 | 1707 | 1546 | 1834 | |
|  | Hispanic | 1182 | 1104 | 1029 | 818 | 1112 | |
|  | NH Black | 1003 | 897 | 862 | 836 | 788 | |
|  | NH White | 2416 | 1978 | 2038 | 1699 | 1778 | |
|  | Another race/ethnicity* | 365 | 340 | 434 | 348 | 360 | |
| *Includes non-Hispanic respondents reporting multiple races. | | | | | | | |

| **Supplemental Table 3. Sociodemographic Characteristics of Female Respondents in the 2008-10 to 2017-19 survey periods of the National Survey of Family Growth** | | | | | |
| --- | --- | --- | --- | --- | --- |
|  | **2008-10** | **2011-13** | **2013-15** | **2015-17** | **2017-19** |
| **Age (y)** |  |  |  |  |  |
| 15-19 | 16.9%  (15.6-18.2%) | 15.7%  (14.1-17.2%) | 15.3% (14.0-16.7%) | 15.3% (13.3-17.3%) | 15.2% (13.7-16.7%) |
| 20-29 | 34.2%  (31.8-36.5%) | 34.1%  (32.5-35.8%) | 34.5%  (32.2-36.8%) | 34.5%  (32.4-36.7%) | 34.4%  (31.2-36.8%) |
| 30-44 | 49.0%  (46.5-51.4%) | 50.2%  (47.8-52.6%) | 50.2%  (47.9-52.5%) | 50.2%  (48.0-52.4%) | 50.4%  (48.2-52.6%) |
| **Race/Ethnicity** |  |  |  |  |  |
| Hispanic/Latino | 17.3% (14.3-20.3%) | 19.7%  (15.9-23.6%) | 20.3% (16.5-24.2%) | 20.8%  (16.9-24.7%) | 21.3% (16.9-25.7%) |
| Non-Hispanic Black, single race | 13.7%  (10.5-17.0%) | 13.9%  (11.4-16.4%) | 13.5%  (10.8-16.2%) | 13.5%  (10.6-16.7%) | 13.6% (10.7-16.5%) |
| Non-Hispanic White, single race | 60.8%  (56.8-64.8%) | 56.9%  (52.9-61.0) | 55.2%  (51.3-59.2%) | 55.2% (50.4-60.1%) | 54.8%  (50.7-58.8%) |
| Another race/ethnicity* | 8.1%  (6.6-9.7%) | 9.4%  (5.5-13.2) | 10.9%  (8.8-13.0%) | 10.3%  (8.8-11.9%) | 10.3%  (8.6-12.1%) |
| **Education** |  |  |  |  |  |
| Less than high school diploma or GED | 24.7%  (23.0-26.5%) | 19.0%  (16.9-21.2%) | 18.3%  (16.6-20.1%) | 18.1% (15.9-20.3%) | 17.0% (15.1-18.9%) |
| High school diploma or GED | 23.2%  (21.7-24.7%) | 25.2%  (23.1-27.2%) | 23.2%  (20.8-25.6%) | 22.5%  (20.6-24.3%) | 22.9%  (20.9-24.9%) |
| Some college or associate degree | 28.8%  (26.3-31.3%) | 30.3%  (27.9-32.7%) | 29.8%  (27.6-31.9%) | 30.6%  (28.0-33.1%) | 30.7%  (29.3-32.2%) |
| Bachelor's degree or higher | 23.2%  (20.8-25.7%) | 25.5%  (22.3-28.6%) | 28.7%  (25.5-31.9%) | 28.9%  (25.4-32.3%) | 29.3%  (26.3-32.3%) |
| **Federal poverty level** |  |  |  |  |  |
| <138% | 33.9%  (31.5-36.4%) | 36.1%  (33.4-38.8%) | 35.7%  (2.9-38.6%) | 33.9% (31.7-36.1%) | 33.1%  (30.2-36.0%) |
| ≥138% | 66.1%  (63.6-68.5%) | 63.9%  (61.2-66.6%) | 64.3%  (61.4-67.1%) | 66.1%  (63.9-68.3%) | 66.9%  (64.0-69.8%) |
| **Health insurance status** |  |  |  |  |  |
| Private only | 60.7%  (57.7-63.6%) | 57.1%  (53.6-60.5%) | 61.6%  (58.2-65.0%) | 61.2%  (58.1-64.3%) | 62.0%  (58.7-65.2%) |
| Any public | 19.0%  (16.8-21.2%) | 22.3%  (19.4-25.2%) | 23.3%  (21.0-25.6%) | 25.3%  (22.3-28.3%) | 25.4%  (22.9-27.9%) |
| None, IHS only, or single service only | 20.3%  (18.5-22.2%) | 20.6%  (18.3-22.9%) | 15.2%  (12.8-17.4%) | 13.5%  (11.4-15.6%) | 12.7%  (10.2-15.1%) |
| **Marital and cohabiting status** | |  |  |  |  |
| Married | 39.4%  (37.0-41.7%) | 38.1%  (35.0-41.2%) | 38.1%  (36.0-40.2%) | 37.8%  (35.4-40.1%) | 35.4%  (33.3-37.5%) |
| Cohabiting, not married | 12.2%  (10.8-13.5%) | 15.0%  (13.5-16.5%) | 14.6%  (13.1-16.1%) | 13.8%  (11.9-15.7%) | 14.7%  (13.2-16.3%) |
| Divorced, widowed, or separated | 9.7%  (8.6-10.8%) | 9.1%  (8.0-10.2%) | 7.6%  (6.7-8.5%) | 7.7% (6.7-8.8%) | 6.5%  (5.6-7.4%) |
| Never married | 37.8%  (36.4-41.2%) | 37.8%  (35.2-40.4%) | 39.7 (37.5-41.9) | 40.7% (37.9-43.6%) | 43.4%  (41.1-45.7%) |
| **Sexual orientation** |  |  |  |  |  |
| Heterosexual or straight | 93.3%  (92.3-94.2%) | 91.8%  (90.7-93.0%) | 90.7% (89.4-91.9%) | 87.4%  (85.8-88.9%) | 83.6%  (81.9-85.3%) |
| Lesbian or gay | 1.4%  (1.0-1.8%) | 1.2%  (0.8-1.6%) | 2.0%  (1.6-2.4%) | 2.3%  (1.5-3.1%) | 2.6%  (2.0-3.2%) |
| Bisexual | 4.3%  (3.7-5.0%) | 5.9%  (5.0-6.7%) | 6.3%  (5.2-7.5%) | 6.6%  (5.8-7.4%) | 10.6%  (9.3-12.0%) |
| Another orientation | n/a | n/a | n/a | 1.7%  (1.1-2.3%) | 2.1%  (1.4-2.8%) |
| Don’t know, Refused, or Not ascertained | 1.0%  (1.7-1.4%) | 1.1%  (0.7-1.5%) | 0.9%  (0.6-1.3%) | 2.1%  (1.2-2.9%) | 1.1%  (0.5-1.6%) |
| GED = General Education Diploma. IHS = Indian Health Service.  *Includes non-Hispanic respondents reporting multiple races.  ^In 2015-17 and 2017-19, respondents were randomized on a 1:1 basis to receive one of two questions regarding sexual orientation: either (1) “Do you think of yourself as… Heterosexual or straight; Homosexual, gay, or lesbian; or Bisexual?” (consistent with prior survey periods) or (2) “Which of the following best represents how you think of yourself? Lesbian or gay; Straight, that is, not lesbian or gay; Bisexual; Something else”. | | | | | |

| **Supplemental Table 4. Sociodemographic Characteristics of Male Respondents in the 2008-10 to 2017-19 survey periods of the National Survey of Family Growth** | | | | | |
| --- | --- | --- | --- | --- | --- |
|  | **2008-10** | **2011-13** | **2013-15** | **2015-17** | **2017-19** |
| **Age (y)** |  |  |  |  |  |
| 15-19 | 17.2% (16.0-18.5%) | 16.7%  (15.3-18.1%) | 16.2%  (14.9-17.6%) | 16.1%  (14.6-17.5%) | 15.9%  (14.2-17.6%) |
| 20-29 | 34.2%  (32.1-36.4%) | 34.4%  (31.8-37.0%) | 34.9%  (32.6-37.1%) | 34.9%  (31.7-38.1%) | 34.8%  (31.0-38.5%) |
| 30-44 | 48.5%  (46.5-50.5%) | 48.9%  (46.7-51.2%) | 48.9%  (46.3-51.5%) | 49.0%  (46.1-52.0%) | 49.3%  (46.0-52.7%) |
| **Race/Ethnicity** |  |  |  |  |  |
| Hispanic/Latino | 19.5%  (16.2-22.8%) | 21.1%  (17.4-24.7%) | 21.4% (17.2-25.7%) | 21.8%  (17.9-25.8%) | 22.3%  (18.1-26.5%) |
| Non-Hispanic Black, single race | 11.9%  (9.1-14.8%) | 11.7%  (9.4-14.1%) | 12.1%  (9.6-14.5%) | 12.3%  (9.7-14.8%) | 12.3%  (10.0-14.7%) |
| Non-Hispanic White, single race | 60.1%  (56-4-63.8%) | 56.7%  (52.8-60.6%) | 56.6%  (52.3-61.0%) | 56.0%  (51.5-60.5%) | 54.7%  (50.3-59.1%) |
| Another race/ethnicity* | 8.5% (6.9-10.0%) | 10.5%  (7.8-13.2%) | 9.9%  (7.6-12.1%) | 9.9%  (8.3-11.4%) | 10.7% (8.5-13.0%) |
| **Education** |  |  |  |  |  |
| Less than high school diploma or GED | 30.9%  (28.6-33.2%) | 22.2%  (20.1-24.2%) | 22.7%  (20.5-24.9%) | 19.4%  (17.7-21.2%) | 17.4% (15.1-19.6%) |
| High school diploma or GED | 23.2%  (21.4-24.9%) | 28.8%  (26.5-31.1%) | 26.8% (24.5-29.2%) | 25.4%  (23.0-27.8%) | 29.2% (26.3-32.1%) |
| Some college or associate degree | 26.3%  (24.3-28.3%) | 27.5%  (24.8-30.1%) | 28.1%  (26.2-30.0%) | 28.0%  (25.6-30.3%) | 27.6% (24.5-30.7%) |
| Bachelor's degree or higher | 19.6%  (17.6-21.7%) | 21.6%  (18.2-25.0%) | 22.4%  (19.6-25.2%) | 27.2%  (23.8-30.6%) | 25.8%  (23.3-28.4%) |
| **Federal poverty level** |  |  |  |  |  |
| <138% | 26.9%  (24.7-29.1%) | 28.1%  (25.4-30.9%) | 26.4%  (24.3-28.4%) | 24.4%  (21.9-26.9%) | 22.6% (20.1-25.0%) |
| ≥138% | 73.1%  (70.9-75.3%) | 71.2%  (69.1-74.6%) | 73.6% (71.6-75.7%) | 75.6%  (73.1-78.1%) | 77.4%  (75.0-79.9%) |
| **Health insurance status** |  |  |  |  |  |
| Private only | 60.4%  (57.5-63.4%) | 57.2%  (53.8-60.6%) | 62.9%  (60.1-65.6%) | 64.9% (61.8-68.1%) | 65.5% (62.0-69.1%) |
| Any public | 13.0%  (11.7-14.3%) | 17.6%  (14.7-20.6%) | 16.9% (14.8-19.0%) | 17.1%  (14.7-19.6%) | 19.2% (16.8-21.5%) |
| None, IHS only, or single service only | 26.5%  (24.0-29.0%) | 25.2%  (22.5-27.8%) | 20.2%  (18.2-22.2%) | 17.9% (15.5-20.3%) | 15.3%  (13.1-17.6%) |
| **Marital and cohabiting status** | |  |  |  |  |
| Married | 36.3%  (34.2-38.4%) | 35.2%  (31.8-38.6%) | 37.0%  (34.6-39.4%) | 35.9% (32.5-39.2% | 34.9% (32.0-37.8%) |
| Cohabiting, not married | 12.6%  (11.2-14.0%) | 13.5%  (11.7-15.3%) | 13.3%  (11.8-14.8%) | 12.3% (10.5-14.1%) | 12.6% (11.0-14.2%) |
| Divorced, widowed, or separated | 5.4%  (4.3-6.5%) | 5.7%  (4.9-6.4%) | 4.6%  (3.9-5.2%) | 3.7% (3.2-4.3%) | 4.0% (3.2-4.8%) |
| Never married | 45.7%  (43.7-47.8%) | 45.6%  (43.1-48.1%) | 45.2%  (42.5-47.8%) | 48.1% (45.2-51.0%) | 48.6% (45.2-51.9%) |
| **Sexual orientation** |  |  |  |  |  |
| Heterosexual or straight | 95.5%  (94.7-96.4%) | 95.2%  (94.3-96.1%) | 95.0%  (94.2-95.8%) | 93.2%  (91.9-94.4%) | 92.2%  (91.2-93.3%) |
| Gay | 1.4%  (0.9-1.8%) | 1.8%  (1.3-2.3%) | 1.7%  (1.2-2.2%) | 2.6%  (1.6-3.6%) | 2.6%  (2.0-3.2%) |
| Bisexual | 1.6%  (1.2-2.1%) | 1.9%  (1.4-2.4%) | 1.9%  (1.4-2.4%) | 2.1%  (1.6-2.6%) | 2.6%  (2.1-3.2%) |
| Another orientation | n/a | n/a | n/a | 1.1%  (0.6-1.5%) | 1.5%  (0.9-2.1%) |
| Don’t know, Refused, or Not ascertained | 1.5%  (1.2-2.1%) | 1.1%  (0.5-1.7%) | 1.4%  (0.9-1.9%) | 1.0%  (0.7-1.4%) | 1.0%  (0.7-1.4%) |
| GED = General Education Diploma. IHS = Indian Health Service.  *Includes non-Hispanic respondents reporting multiple races.  ^In 2015-17 and 2017-19, respondents were randomized on a 1:1 basis to receive one of two questions regarding sexual orientation: either (1) “Do you think of yourself as… Heterosexual or straight; Homosexual or gay; or Bisexual?” (consistent with prior survey periods) or (2) “Which of the following best represents how you think of yourself? Gay; Straight, that is, not gay; Bisexual; Something else”. | | | | | |

| **Supplemental Table 5. Sexual behaviors and STI testing, treatment, and diagnosis among female respondents, stratified by age, in the 2008-10 to 2017-19 survey periods of the National Survey of Family Growth** | | | | | | | | |
| --- | --- | --- | --- | --- | --- | --- | --- | --- |
|  | **Age** | **% or Mean (95% Confidence Interval)** | | | | | **Logistic or linear regression** | |
|  |  | **2008-10** | **2011-13** | **2013-15** | **2015-17** | **2017-19** | **OR or β (95%CI)** | **p-value** |
| *Sexual behaviors* |  |  |  |  |  |  |  |  |
| Ever had vaginal sex with male sex partner | 15-19 | 45.3% (41.4-49.3%) | 44.8% (39.3-50.3%) | 41.0% (36.3-45.7%) | 42.0% (36.0-48.0%) | 41.5% (37.0-46.0%) | 0.981 (0.955, 1.007) | 0.143 |
|  | 20-29 | 91.5% (89.3-93.7%) | 91.2% (89.2-93.1%) | 91.5% (89.7-93.3%) | 88.9% (85.5-92.3%) | 87.9% (85.7-90.0%) | **0.953 (0.918, 0.990)** | **0.013** |
|  | 30-44 | 98.4% (97.8-99.0%) | 98.7% (98.2-99.2%) | 98.5% (97.9-99.1%) | 98.3% (97.7-99.0%) | 98.5% (97.7-99.2%) | 0.992 (0.932, 1.056) | 0.800 |
| Vaginal sex with male sex partner(s) in past 12 months | 15-19 | 92.4% (90.0-94.7%) | 89.0% (85.4-92.6%) | 92.2% (88.7-95.7%) | 93.5% (90.8-96.2%) | 89.4% (84.7-94.1%) | 0.989 (0.930, 1.052) | 0.735 |
|  | 20-29 | 93.9% (92.7-95.2%) | 92.6% (90.7-94.6%) | 90.2% (88.3-92.1%) | 94.6% (93.5-95.6%) | 89.7% (87.4-91.9%) | **0.958 (0.926, 0.992)** | **0.015** |
|  | 30-44 | 89.6% (87.9-91.2%) | 88.0% (86.3-89.7%) | 89.6% (88.0-91.3%) | 89.4% (87.8-90.9%) | 89.0% (87.2-90.9%) | 1.001 (0.976, 1.026) | 0.957 |
| Number of male sex partners in past 12 months* | 15-19 | 1.75 (1.58-1.92) | 1.60 (1.40-1.79) | 1.71 (1.55-1.88) | 1.62 (1.41-1.83) | 1.73 (1.51-1.95) | -0.003 (-0.031, 0.025) | 0.830 |
|  | 20-29 | 1.35 (1.29-1.41) | 1.35 (1.27-1.44) | 1.32 (1.26-1.38) | 1.43 (1.35-1.51) | 1.37 (1.29-1.46) | 0.006 (-0.005, 0.016) | 0.285 |
|  | 30-44 | 1.18 (1.06-1.30) | 1.13 (1.09-1.16) | 1.12 (1.10-1.14) | 1.14 (1.10-1.17) | 1.10 (1.08-1.13) | -0.007 (-0.019, 0.010) | 0.274 |
| Condom use at last vaginal sex | 15-19 | 58.0% (51.1-64.9%) | 55.9% (48.1-63.7%) | 59.0% (51.7-66.3%) | 53.5% (46.3-60.7%) | 52.8% (43.4-62.2%) | 0.977 (0.934, 1.023) | 0.372 |
|  | 20-29 | 33.6% (30.0-37.2%) | 30.3% (27.0-33.7%) | 29.% (25.1-33.1%) | 27.0% (23.6-30.3%) | 27.9% (24.0-31.8%) | **0.967 (0.943, 0.992)** | **0.010** |
|  | 30-44 | 17.7% (15.2-20.1%) | 18.3% (16.1-20.6%) | 17.6% (15.5-19.8%) | 17.9% (14.3-21.4%) | 17.9% (15.3-20.6%) | 1.001 (0.975, 1.026) | 0.967 |
| ≥1 vaginal sex act in past 4 weeks | 15-19 | 66.1%  (61.3-71.0%) | 61.5%  (54.3-68.7%) | 64.2%  (58.7-69.7%) | 70.5%  (61.7-79.2%) | 61.1%  (53.8-68.5%) | 0.997  (0.958, 1.037) | 0.878 |
|  | 20-29 | 82.8%  (80.4-85.2%) | 83.7%  (81.2-86.1%) | 82.6%  (79.7-85.4%) | 84.0%  (81.5-86.5%) | 83.2%  (79.9-86.5%) | 1.004  (0.976, 1.033) | 0.774 |
|  | 30-44 | 85.2%  (83.2-87.1%) | 83.5%  (81.0-85.9%) | 85.1%  (82.9-87.2%) | 83.7%  (81.1-86.3%) | 86.5%  (84.6-88.4%) | 1.009  (0.986, 1.032) | 0.443 |
| Number of vaginal sex acts in past 4 weeks* | 15-19 | 6.35 (5.66-7.03) | 6.51 (5.41-7.62) | 6.32 (5.06-7.58) | 4.79 (3.89-5.69) | 5.79 (4.57-7.02) | -0.128 (-0.262. 0.010) | 0.064 |
|  | 20-29 | 8.35 (7.59-9.12) | 8.50 (7.84-9.17) | 8.26 (7.60-8.95) | 7.84 (7.26-8.42) | 7.38 (6.94-7.82) | **-0.111 (-0.200, -0.020)** | **0.020** |
|  | 30-44 | 6.85 (6.39-7.31) | 6.96 (6.47-7.44) | 6.63 (6.21-7.05) | 6.82 (6.25-7.39) | 6.76 (6.24-7.28) | -0.014 (-0.085, 0.060) | 0.704 |
| Proportion of condom-protected vaginal sex acts in past 4 weeks* | 15-19 | 0.526 (0.452-0.599) | 0.536 (0.460-0.613) | 0.546 (0.472-0.620) | 0.505 (0.397-0.613) | 0.501 (0.388-0.613) | -0.003 (-0.016, 0.010) | 0.641 |
|  | 20-29 | 0.343 (0.309-0.378) | 0.275 (0.243-0.307) | 0.253 (0.215-0.291) | 0.234 (0.205-0.263) | 0.250 (0.206-0.294) | **-0.011 (-0.01, 0.006)** | **<0.0001** |
|  | 30-44 | 0.164 (0.140-0.188) | 0.153 (0.126-0.180) | 0.152 (0.130-0.174) | 0.159 (0.125-0.193) | 0.143 (0.120-0.166) | -0.002 (-0.005, 0.002) | 0.342 |
| Vaginal, oral, or anal sex with a man who has sex with men in past 12 months | 15-19 | 3.5% (1.5-5.5%) | 2.5% (0.8-4.2%) | 2.5% (0.4-4.5%) | 3.6% (1.3-5.8%) | 3.5% (0.0-7.4%) | 1.009 (0.886, 1.149) | 0.890 |
|  | 20-29 | 1.9% (1.2-2.8%) | 1.7% (0.8-2.7%) | 3.0% (1.9-4.1%) | 2.8% (1.1-4.6%) | 3.5% (1.8-5.2%) | **1.080 (1.004, 1.163)** | **0.037** |
|  | 30-44 | 1.7% (0.9-2.6%) | 1.8% (0.9-2.7%) | 1.3% (0.8%-1.8%) | 1.2% (0.8-1.6%) | 2.3% (1.5-3.1%) | 1.013  (0.943, 1.088) | 0.717 |
| Racial/ethnic homophily among up to 3 current partners with whom vaginal sex occurred most recently^ | 15-19 | 87.6%  (81.9-93.4%) | 74.9%  (65.0-84.8%) | 73.0%  (64.2-81.7%) | 80.1%  (70.5-89.6%) | 76.1%  (68.0-84.1%) | 0.952  (0.891, 1.017) | 0.147 |
|  | 20-29 | 84.3%  (81.6-87.1%) | 80.7%  (77.6-83.9%) | 82.1%  (78.9-85.2%) | 80.7%  (75.9-85.5%) | 78.6%  (74.3-82.8%) | **0.965**  **(0.933, 0.998)** | **0.040** |
|  | 30-44 | 88.9%  (86.6-91.1%) | 86.3%  (83.7-89.0%) | 85.8%  (83.6-87.9%) | 86.9%  (84.0-89.8%) | 87.0%  (84.2-89.8%) | 0.985  (0.953, 1.017) | 0.358 |
| Concurrency (≥2 current partners at time of survey) | 15-19 | 2.4% (0.5-4.2%) | 0.4% (0.0-0.8%)^‡^ | 0.8% (0.0-2.0%)^‡^ | 2.2% (0.0-4.5%)^‡^ | 0.5% (0.0-1.0%)^‡^ | 0.915 (0.760, 1.102) | 0.361 |
|  | 20-29 | 1.3% (0.7-1.8%) | 0.9% (0.4-1.5%) | 0.6% (0.3-1.0%) | 1.7% (0.4-2.9%) | 1.4% (0.5-2.3%) | 1.035 (0.943, 1.135) | 0.467 |
|  | 30-44 | 0.7% (0.3-1.0%) | 1.5% (0.8-2.1%) | 0.8% (0.5-1.2%) | 1.0% (0.5-1.6%) | 1.3% (0.7-1.9%) | 1.043 (0.972, 1.119) | 0.237 |
|  |  |  |  |  |  |  |  |  |
| *STI Testing, Treatment, and Diagnosis* |  |  |  |  |  |  |  |  |
| Chlamydia testing in past 12 months | 15-19 | 40.7% (34.9-46.5%) | 34.7% (27.6-41.8%) | 36.8% (30.8-42.9%) | 33.5% (25.6-41.5%) | 32.1% (24.7-39.4%) | 0.962 (0.923, 1.004) | 0.073 |
|  | 20-29 | 38.9% (35.3-42.5%) | 38.7% (34.6-42.9%) | 43.1% (39.8-46.3%) | 44.2% (40.9-47.5%) | 41.7% (37.7-45.6%) | 1.021 (0.999, 1.044) | 0.061 |
|  | 30-44 | 15.5% (13.5-17.4%) | 19.1% (16.6-21.6%) | 21.7% (18.7-24.7%) | 23.5% (20.6-26.4%) | 22.6% (20.1-25.0%) | **1.055 (1.034, 1.077)** | **<0.0001** |
| STI testing in past 12 months† | 15-19 | NA | 38.2% (31.0-45.3%) | 41.8% (35.2-48.3%) | 38.5% (29.8-47.2%) | 38.3% (30.5-46.0%) | 0.994 (0.927, 1.067) | 0.875 |
|  | 20-29 | NA | 45.4% (40.7-50.0%) | 49.7% (46.3-53.1%) | 50.8% (47.7-53.9%) | 47.8% (43.8-51.9%) | 1.018 (0.979, 1.057) | 0.369 |
|  | 30-44 | NA | 22.8% (20.4-25.3%) | 26.3% (22.9-29.7%) | 29.0% (25.9-32.1%) | 27.7% (25.0-30.4%) | **1.045 (1.013, 1.078)** | **0.006** |
| STI treatment in past 12 months† | 15-19 | NA | 19.7% (11.2-28.1%) | 23.4% (13.6-33.3%) | 20.7% (9.4-32.0%) | 23.0% (15.4-30.6%) | 1.021 (0.914, 1.141) | 0.708 |
|  | 20-29 | NA | 14.9% (11.0-18.8%) | 13.8% (10.8-16.8%) | 12.5% (9.3-15.7%) | 14.5% (10.3-18.6%) | 0.989 (0.918, 1.064) | 0.758 |
|  | 30-44 | NA | 10.0% (6.3-13.7%) | 9.5% (6.5-12.4%) | 8.2% (4.5-11.9%) | 11.0% (8.2-13.9%) | 1.013 (0.931, 1.101) | 0.765 |
| Gonorrhea diagnosis in past 12 months† | 15-19 | NA | 1.7% (0.2-3.1%) | 2.4% (0.0-4.9%) | 2.5% (0.0-5.8%) | 9.8% (4.3-15.2%) | **1.411 (1.131, 1.761)** | **0.002** |
|  | 20-29 | NA | 2.8% (1.4-4.1%) | 1.8% (0.8-2.8%) | 2.4% (1.0-3.8%) | 2.9% (1.3-4.4%) | 1.021 (0.895, 1.165) | 0.759 |
|  | 30-44 | NA | 1.3% (0.4-2.1%) | 1.3% (0.6-2.0%) | 1.3% (0.3-2.3%) | 2.0% (1.0-3.0%) | 1.088 (0.944, 1.254) | 0.242 |
| Chlamydia diagnosis in past 12 months† | 15-19 | NA | 10.9% (3.8-18.0%) | 16.1% (7.7-24.5%) | 14.0% (4.8-23.2%) | 16.3% (9.4-23.1%) | 1.059 (0.926, 1.211) | 0.401 |
|  | 20-29 | NA | 5.5% (3.9-7.1%) | 4.5% (3.0-6.0%) | 7.2% (4.2-10.2%) | 5.9% (3.4-8.4%) | 1.038 (0.953, 1.131) | 0.392 |
|  | 30-44 | NA | 1.8% (0.7-2.8%) | 1.9% (0.8-2.9%) | 2.4% (0.0-5.2%) | 1.5% (0.6-2.4%) | 0.993 (0.864, 1.141) | 0.917 |
| Bold indicates significance at <0.05 level.  *Estimates derived from linear regression (β). All others from logistic regression (odds ratios; OR). All models accounted for survey weights.  ^Measured among Hispanic, Non-Hispanic Black, and Non-Hispanic White respondents only.  †Analysis includes 2011-13 through 2017-19 survey periods only. In the 2008-10 survey periods, female respondents were not asked about STI testing other than chlamydia and were not asked about gonorrhea or chlamydia diagnoses unless they reported STI treatment in the past 12 months.  ^‡^Estimate based on numerator fewer than 5 cases or denominator fewer than 100 cases, and therefore does not meet standards of reliability or precision (Copen, Chandra, & Febo-Vazquez, 2016). | | | | | | | | |

| **Supplemental Table 6. Sexual behaviors and STI testing, treatment, and diagnosis among male respondents, stratified by age, in the 2008-10 to 2017-19 survey periods of the National Survey of Family Growth** | | | | | | | | |
| --- | --- | --- | --- | --- | --- | --- | --- | --- |
|  | **Age** | **% or Mean (95% Confidence Interval)** | | | | | **Logistic or linear regression** | |
|  |  | **2008-10** | **2011-13** | **2013-15** | **2015-17** | **2017-19** | **OR or β (95%CI)** | **p-value** |
| *Sexual behaviors* |  |  |  |  |  |  |  |  |
| Ever had vaginal sex with female sex partner | 15-19 | 42.2%  (38.6-45.7%) | 47.1%  (42.8-51.5%) | 42.1%  (38.3-45.9%) | 37.8%  (32.0-43.6%) | 40.2%  (35.5-44.8%) | 0.978  (0.954, 1.003) | 0.085 |
|  | 20-29 | 89.3%  (87.3-91.3%) | 89.8%  (87.6-92.0%) | 90.6%  (88.8-92.4%) | 88.6%  (86.1-91.1%) | 86.3%  (81.8-90.9%) | 0.970  (0.925, 1.017) | 0.200 |
|  | 30-44 | 98.4%  (97.7-99.1%) | 97.8%  (96.6-98.9%) | 98.3%  (97.7-98.9%) | 97.3%  (96.1-98.4%) | 97.1%  (96.0-98.1%) | **0.937**  **(0.881-0.997)** | **0.039** |
| Vaginal sex with male sex partner(s) in past 12 months | 15-19 | 91.8%  (89.1-94.5%) | 90.7%  (88.2-93.1%) | 87.6%  (83.4-91.9%) | 87.7%  (82.8-92.6%) | 88.3%  (84.0-92.6%) | 0.951  (0.902, 1.003) | 0.065 |
|  | 20-29 | 90.1%  (88.0-92.1%) | 89.9%  (87.8-91.9%) | 89.9%  (88.0-91.9%) | 87.1%  (84.6-89.7%) | 89.6%  (87.3-92.0%) | 0.983  (0.951, 1.016) | 0.304 |
|  | 30-44 | 91.5%  (90.0-92.9%) | 91.2%  (89.8-92.7%) | 92.1%  (90.2-94.0%) | 93.0%  (91.5-94.5%) | 91.0%  (89.2-92.7%) | 1.005  (0.975, 1.035) | 0.761 |
| Number of female sex partners in past 12 months* | 15-19 | 1.91  (1.76-2.06) | 1.99  (1.78-2.21) | 1.89  (1.70-2.08) | 1.79  (1.64-1.95) | 1.60  (1.45-1.74) | **-0.035**  **(-0.057, -0.013)** | **0.002** |
|  | 20-29 | 1.65  (1.57-1.72) | 1.65  (1.54-1.77) | 1.65  (1.53-1.76) | 1.58  (1.47-1.69) | 1.52  (1.43-1.61) | **-0.014**  **(-0.027, -0.002)** | **0.027** |
|  | 30-44 | 1.26  (1.21-1.30) | 1.25  (1.20-1.30) | 1.21  (1.16-1.25) | 1.24  (1.19-1.29) | 1.21  (1.17-1.25) | -0.004  (-0.011, 0.002) | 0.192 |
| Condom use at last vaginal sex | 15-19 | 72.4%  (66.8-77.9%) | 76.2%  (70.5-82.0%) | 73.6%  (68.3-78.9%) | 65.7%  (58.0-73.5%) | 70.2%  (64.0-76.4%) | 0.971  (0.930, 1.013) | 0.174 |
|  | 20-29 | 44.2%  (40.8-47.6%) | 44.9%  (41.1-48.8%) | 44.7%  (40.9-48.5%) | 43.1%  (38.6-47.6%) | 38.6%  (33.9-43.4%) | 0.978  (0.955, 1.001) | 0.063 |
|  | 30-44 | 22.7%  (20.2-25.3%) | 24.9%  (21.8-28.1%) | 21.8%  (19.0-24.7%) | 22.1%  (18.9-25.3%) | 22.2%  (19.8-24.6%) | 0.991  (0.971, 1.013) | 0.419 |
| ≥1 vaginal sex act in past 4 weeks | 15-19 | 62.0%  (55.8-68.1%) | 61.0%  (53.5-68.4%) | 57.4%  (51.1-63.8%) | 56.4%  (49.2-63.5%) | 61.5% (54.2-68.7%) | 0.988  (0.949, 1.029) | 0.564 |
|  | 20-29 | 80.2% (77.0-83.4%) | 80.2%  (77.6-82.7%) | 79.4% (76.5-82.4%) | 79.2%  (75.3-83.2%) | 74.5%  (70.1-78.9%) | 0.969  (0.938, 1.000) | 0.052 |
|  | 30-44 | 85.7%  (82.9-88.4%) | 85.0%  (82.7-87.3%) | 85.2%  (82.8-87.7%) | 85.1%  (82.5-87.6%) | 83.2%  (80.4-85.9%) | 0.983  (0.954, 1.014) | 0.272 |
| Number of vaginal sex acts in past 4 weeks* | 15-19 | 3.91 (2.96-4.85) | 3.26  (2.61-3.92) | 2.90  (2.23-3.57) | 3.01  (2.11-3.92) | 2.68  (2.14-3.21) | -0.127  (-0.240, -0.014) | 0.028 |
|  | 20-29 | 6.20  (5.59-6.80) | 6.62  (5.91-7.32) | 6.44  (5.82-7.07) | 5.96 (5.29-6.64) | 5.10  (4.48-5.71) | **-0.119**  **(-0.209, -0.028)** | **0.011** |
|  | 30-44 | 5.61  (5.01-6.21) | 6.12  (5.74-6.50) | 6.08  (5.59-6.57) | 5.88  (5.47-6.29) | 5.45  (4.99-5.90) | -0.018  (-0.093, 0.056) | 0.632 |
| Proportion of condom-protected vaginal sex acts in past 4 weeks* | 15-19 | 0.708  (0.645-0.772) | 0.716  (0.655-0.777) | 0.718  (0.643-0.792) | 0.651  (0.550-0.752) | 0.649  (0.556-0.743) | -0.008  (-0.019, 0.004) | 0.185 |
|  | 20-29 | 0.400  (0.365-0.436) | 0.409  (0.359-0.458) | 0.385  (0.343-0.428) | 0.354  (0.314-0.394) | 0.336  (0.297-0.375) | **-0.008**  **(-0.013, -0.002)** | **0.005** |
|  | 30-44 | 0.179  (0.152-0.205) | 0.179  (0.149-0.210) | 0.178  (0.147-0.209) | 0.164  (0.137-0.190) | 0.178  (0.151-0.205) | -0.001  (-0.005, 0.003) | 0.694 |
| Ever had oral or anal sex with another man | 15-19 | 3.0%  (0.9-5.2%) | 1.8%  (0.7-3.0%) | 1.4%  (0.4-2.3%) | 4.0%  (1.5-6.6%) | 2.8%  (1.0-4.6%) | 1.020  (0.907, 1.148) | 0.737 |
|  | 20-29 | 4.5%  (2.3-6.6%) | 4.3%  (2.9-5.8%) | 3.3%  (2.2-4.4%) | 4.3%  (2.4-6.2%) | 5.2%  (3.8-6.7%) | 1.014  (0.950, 1.082) | 0.679 |
|  | 30-44 | 3.0%  (2.1-3.9% | 4.5%  (3.1-5.9%) | 3.9%  (2.8-5.1%) | 4.3%  (2.9-5.7%) | 5.2%  (3.9%-6.5%) | **1.052 (1.008, 1.097)** | **0.019** |
| Racial/ethnic homophily, among up to 3 current partners with whom vaginal sex occurred most recently^ | 15-19 | 76.2%  (69.4-82.9%) | 82.7%  (77.2-88.2%) | 74.0%  (65.5-82.5%) | 74.5%  (66.2-82.8%) | 68.2%  (58.0-78.4%) | 0.948  (0.889, 1.011) | 0.101 |
|  | 20-29 | 85.6%  (82.8-88.5%) | 80.4%  (76.9-83.8%) | 81.1%  (76.5-85.7%) | 77.1%  (72.8-81.4%) | 75.8%  (71.8-79.7%) | **0.934**  **(0.905, 0.965)** | **<0.0001** |
|  | 30-44 | 87.5%  (85.5-89.5%) | 84.5%  (81.3-87.7%) | 84.7%  (82.0-87.5%) | 81.2%  (77.1-85.2%) | 84.3%  (81.4-87.3%) | **0.965**  **(0.937, 0.994)** | **0.020** |
| Concurrency (≥2 current partners at time of survey) | 15-19 | 2.7%  (1.3-4.1%) | 2.7%  (1.2-4.1%) | 4.2%  (1.8-6.5%) | 1.8%  (0.0-3.6%) | 2.1%  (0.4-3.7%) | 0.970  (0.890, 1.057) | 0.481 |
|  | 20-29 | 3.0%  (1.7-4.3%) | 2.2%  (1.2-3.1%) | 3.0%  (1.8-4.1%) | 1.8%  (0.8-2.9%) | 1.4% (0.5-2.3%) | **0.932**  **(0.869, 1.000)** | **0.049** |
|  | 30-44 | 2.7%  (1.4-4.0%) | 2.5%  (1.7-3.4%) | 2.1%  (1.2-3.0%) | 2.2%  (1.1-3.3%) | 1.5%  (0.8-2.1%) | 0.946  (0.885, 1.011) | 0.099 |
|  |  |  |  |  |  |  |  |  |
| *STI Testing, Treatment, and Diagnosis* |  |  |  |  |  |  |  |  |
| STI testing in past 12 months | 15-19 | 20.5%  (16.0-24.9%) | 20.4%  (16.6-24.3%) | 17.0%  (11.6-22.4%) | 16.5%  (11.4-21.7%) | 15.4%  (11.0-19.9%) | 0.959  (0.918, 1.001) | 0.058 |
|  | 20-29 | 23.5%  (19.5-27.4%) | 24.4%  (21.0-27.9%) | 21.2%  (18.3-24.2%) | 21.2%  (17.7-24.8%) | 18.6%  (14.6-22.6%) | **0.968**  **(0.937, 1.000)** | **0.050** |
|  | 30-44 | 11.9%  (10.1-13.7%) | 12.2%  (9.9-14.6%) | 13.0%  (10.6-15.3%) | 14.5%  (12.1-16.8%) | 12.8%  (10.5-15.1%) | 1.016  (0.989, 1.044) | 0.233 |
| STI treatment in past 12 months | 15-19 | 15.3%  (6.0-24.6%) | 25.6%  (12.1-39.1%) | 26.9%  (14.0-39.8%)^‡^ | 13.2%  (4.5-22.0%)^‡^ | 9.3%  (1.9-16.8%)^‡^ | 0.819  (0.694, 0.966) | 0.018 |
|  | 20-29 | 19.3%  (12.8-25.9%) | 11.3%  (7.9-14.7%) | 16.2%  (10.3-22.2%) | 15.4%  (7.2-23.6%) | 13.6%  (9.3-17.9%) | 1.031  (0.951, 1.117) | 0.462 |
|  | 30-44 | 11.2%  (6.6-15.8%) | 8.3%  (3.8-12.7%) | 8.4%  (5.0-11.9%) | 12.3%  (5.7-19.0%) | 8.1%  (4.5-11.6%) | 1.021  (0.914, 1.142) | 0.709 |
| Gonorrhea diagnosis in past 12 months† | 15-19 | NA | 4.5%  (0.4-8.5%) | 2.9%  (0.0-6.8%)^‡^ | 6.6%  (0.0-14.4%)^‡^ | 4.9%  (0.0-10.5%)^‡^ | 1.052  (0.821, 1.347) | 0.689 |
|  | 20-29 | NA | 2.1%  (0.6-3.6%) | 2.8%  (1.0-4.6%) | 2.5%  (0.8-4.1%) | 1.9%  (0.2-3.6%) | 0.985  (0.839, 1.156) | 0.851 |
|  | 30-44 | NA | 2.0%  (0.4-3.5%) | 1.5%  (0.1-3.0%) | 3.0%  (0.0-6.0%) | 1.9%  (0.3-3.5%) | 1.033  (0.862, 1.238) | 0.723 |
| Chlamydia diagnosis in past 12 months† | 15-19 | NA | 5.2%  (1.3-9.1%) | 16.5%  (2.1-30.9%)^‡^ | 7.8%  (0.0-16.0%)^‡^ | 8.2%  (0.6-15.9%)^‡^ | 1.032  (0.873, 1.221) | 0.711 |
|  | 20-29 | NA | 4.6%  (2.7-6.6%) | 5.6%  (2.0-9.2%) | 9.1%  (2.1-16.0%) | 4.4%  (1.5-7.3%) | 1.035  (0.921, 1.164) | 0.558 |
|  | 30-44 | NA | 2.4%  (0.7-4.1%) | 3.2%  (1.1-5.3%) | 2.9%  (0.3-5.6%) | 1.5%  (0.0-3.0%) | 0.940  (0.801, 1.103) | 0.447 |
| Bold indicates significance at <0.05 level.  *Estimates derived from linear regression (β). All others from logistic regression (odds ratios; OR). All models accounted for survey weights.  ^Measured among Hispanic, Non-Hispanic Black, and Non-Hispanic White respondents only. †Analysis includes 2011-13 through 2017-19 survey periods only. In the 2008-10 survey period, male respondents were not asked about gonorrhea or chlamydia diagnoses unless they reported STI treatment in the past 12 months.  ^‡^Estimate based on numerator fewer than 5 cases or denominator fewer than 100 cases, and therefore does not meet standards of reliability or precision (Copen et al., 2016). | | | | | | | | |

| **Supplemental Table 7. Sexual behaviors and STI testing, treatment, and diagnosis among female respondents, stratified by race/ethnicity, in the 2008-10 to 2017-19 survey periods of the National Survey of Family Growth** | | | | | | | | |
| --- | --- | --- | --- | --- | --- | --- | --- | --- |
|  | **Race / Ethnicity** | **% or Mean (95% Confidence Interval)** | | | | | **Logistic or linear regression** | |
|  |  | **2008-10** | **2011-13** | **2013-15** | **2015-17** | **2017-19** | **OR or β (95%CI)** | **p-value** |
| *Sexual behaviors* |  |  |  |  |  |  |  |  |
| Ever had vaginal sex with male sex partner | Hispanic | 86.8%  (85.1-88.6%) | 87.8%  (85.3-90.2%) | 85.7%  (83.2-88.3%) | 86.2%  (82.9-89.5%) | 82.2%  (79.7-84.8%) | **0.961**  **(0.935, 0.987)** | **0.004** |
|  | NH Black | 87.5%  (85.1-89.9%) | 88.2%  (85.8-90.6%) | 88.7%  (86.3-91.1%) | 89.2%  (85.8-92.6%) | 88.0%  (85.0-91.0%) | 1.010  (0.972, 1.049) | 0.624 |
|  | NH White | 87.9%  (85.7-90.1%) | 88.6%  (86.5-90.7%) | 88.3%  (86.8-89.8%) | 86.0%  (83.8-88.2%) | 87.4% (85.6-89.2%) | 0.986  (0.959, 1.014) | 0.320 |
| Vaginal sex with male sex partner(s) in past 12 months | Hispanic | 91.2%  (89.0-93.3%) | 87.9%  (85.4-90.4%) | 91.2%  (89.2-93.2%) | 91.7%  (89.0-94.3%) | 90.4%  (88.3-92.5%) | 1.011  (0.974, 1.049) | 0.576 |
|  | NH Black | 89.2%  (86.7-91.6%) | 86.2%  (82.4-90.1%) | 87.9%  (84.8-90.9%) | 88.5%  (85.7-91.2%) | 87.3%  (84.0-90.7%) | 0.992  (0.953, 1.032) | 0.690 |
|  | NH White | 91.9%  (90.6-93.2%) | 91.5%  (89.6-93.4%) | 90.6%  (88.9-92.4%) | 92.5%  (90.9-94.1%) | 89.6%  (87.7-91.5%) | 0.980  (0.953, 1.009) | 0.171 |
| Number of male sex partners in past 12 months* | Hispanic | 1.14  (1.09-1.18) | 1.24  (1.12-1.37) | 1.20  (1.13-1.26) | 1.17  (1.12-1.23) | 1.16  (1.12-1.21) | 0.000  (-0.008, 0.008) | 0.953 |
|  | NH Black | 1.36  (1.27-1.44) | 1.32  (1.25-1.39) | 1.24  (1.19-1.30) | 1.35  (1.27-1.42) | 1.27  (1.17-1.37) | -0.007  (-0.019, 0.005) | 0.271 |
|  | NH White | 1.32  (1.21-1.43) | 1.23  (1.17-1.29) | 1.25  (1.21-1.28) | 1.30  (1.25-1.35) | 1.28  (1.21-1.34) | -0.002  (-0.014, 0.011) | 0.808 |
| Condom use at last vaginal sex | Hispanic | 28.9%  (25.3-32.6%) | 27.0%  (23.5-30.4%) | 27.0% (23.1-30.9%) | 24.7%  (19.8-29.6%) | 27.9%  (23.2-32.6%) | 0.990  (0.960, 1.020) | 0.506 |
|  | NH Black | 41.7%  (37.8-45.6%) | 35.8% (31.2-40.4%) | 35.0%  (30.4-39.6%) | 28.4%  (24.2-32.6%) | 37.9%  (32.4-43.4%) | **0.968**  **(0.941, 0.996)** | **0.027** |
|  | NH White | 22.7%  (19.7-25.6%) | 22.6%  (20.0-25.2%) | 21.4%  (18.8-24.0%) | 21.3%  (17.8-24.8%) | 18.9%  (16.2-21.7%) | 0.978  (0.954, 1.002) | 0.075 |
| ≥1 vaginal sex act in past 4 weeks | Hispanic | 83.3% (80.2-86.4%) | 78.1%  (75.0-81.1%) | 81.9%  (78.4-85.5%) | 83.2%  (79.5-86.9%) | 83.7%  (80.6-86.8%) | 1.016  (0.986, 1.048) | 0.295 |
|  | NH Black | 74.5%  (71.2-77.8%) | 73.2%  (69.5-77.0%) | 71.6%  (67.9-75.4%) | 74.4%  (67.9-80.9%) | 78.9%  (74.8-83.0%) | 1.021  (0.989, 1.054) | 0.200 |
|  | NH White | 84.3%  (82.1-86.5%) | 85.2%  (82.8-87.5%) | 85.5%  (83.2-87.9%) | 86.0%  (84.0-87.9%) | 84.9%  (82.7-87.2%) | 1.009  (0.984, 1.034) | 0.493 |
| Number of vaginal sex acts in past 4 weeks* | Hispanic | 7.15  (6.74-7.56) | 7.72  (7.02-8.43) | 7.41  (6.88-7.95) | 7.36  (6.69-8.10) | 6.77  (6.17-7.38) | -0.045  (-0.122, 0.033) | 0.259 |
|  | NH Black | 7.25  (6.61-7.89) | 7.14  (6.36-7.92) | 6.32  (5.42-7.22) | 6.54  (5.73-7.35) | 6.25  (5.38-7.13) | **-0.117**  **(-0.225, -0.009)** | **0.035** |
|  | NH White | 7.44  (6.96-7.92) | 7.51  (7.02-8.00) | 7.41  (6.92-7.90) | 6.96  (6.44-7.49) | 7.04  (6.58-7.49) | -0.059  (-0.127, 0.010) | 0.093 |
| Proportion of condom-protected vaginal sex acts in past 4 weeks* | Hispanic | 0.297  (0.253-0.342) | 0.237  (0.195-0.279) | 0.230  (0.192-0.269) | 0.237  (0.190-0.285) | 0.240  (0.190-0.290) | -0.005  (-0.012, 0.002) | 0.129 |
|  | NH Black | 0.353  (0.314-0.392) | 0.294  (0.245-0.343) | 0.299  (0.252-0.345) | 0.219  (0.172-0.266) | 0.310  (0.242-0.378) | **-0.008**  **(-0.015, 0.000)** | **0.044** |
|  | NH White | 0.222  (0.194-0.251) | 0.197  (0.175-0.220) | 0.192  (0.165-0.219) | 0.199 (0.163-0.234) | 0.162  (0.139-0.185) | **-0.005**  **(-0.009, -0.002)** | **0.007** |
| Vaginal, oral, or anal sex with a man who has sex with men in past 12 months | Hispanic | 2.2%  (1.0-3.4%) | 1.9%  (1.1-2.8%) | 2.1%  (1.1-3.1%) | 1.7%  (0.7-2.8%) | 3.0%  (1.1-4.9%) | 1.029  (0.934, 1.134) | 0.560 |
|  | NH Black | 1.7%  (0.8-2.7%) | 0.9%  (0.1-1.7%) | 3.2%  (1.8-4.5%) | 1.7%  (0.8-2.6%) | 3.5%  (1.5-5.5%) | 1.093  (0.993, 1.205) | 0.071 |
|  | NH White | 1.8%  (0.9-2.6%) | 1.9%  (1.0-2.8%) | 1.8%  (1.1-2.5%) | 2.2%  (1.0-3.4%) | 2.7%  (1.5-4.0%) | 1.051  (0.977, 1.130) | 0.180 |
| Racial/ethnic homophily among up to 3 current partners with whom vaginal sex occurred most recently | Hispanic | 80.5%  (76.4-84.6%) | 72.0%  (66.2-77.7%) | 71.5%  (65.9-77.2%) | 79.7%  (74.1-85.3%) | 76.7%  (70.9-82.5%) | 0.999  (0.958, 1.041) | 0.962 |
|  | NH Black | 90.8%  (87.6-94.0%) | 90.4%  (87.7-93.2%) | 89.2%  (85.6-92.8%) | 92.0%  (89.0-95.0%) | 84.8%  (78.7-90.9%) | 0.952  (0.892, 1.017) | 0.142 |
|  | NH White | 88.2%  (86.1-90.3%) | 85.7%  (83.3-88.2%) | 86.7%  (84.1-89.3%) | 84.1%  (80.5-87.7%) | 85.5%  (83.3-87.7%) | 0.972  (0.945, 1.000) | 0.051 |
| Concurrency (≥2 current partners at time of survey) | Hispanic | 0.1%  (0.0-0.2%)^‡^ | 0.7%  (0.2-1.2%) | 0.6%  (0.1-1.0%) | 1.1%  (0.1-2.0%) | 0.4%  (0.1-0.7%) | 1.093  (0.993, 1.203) | 0.070 |
|  | NH Black | 3.3%  (2.1-4.5%) | 3.3%  (1.7-5.0%) | 1.7%  (0.6-2.7%) | 2.1%  (1.0-3.2%) | 1.5%  (0.5-2.6%) | **0.913**  **(0.852, 0.979)** | **0.013** |
|  | NH White | 0.8%  (0.4-1.2%) | 1.0%  (0.4-1.5%) | 0.5%  (0.3-0.7%) | 1.3%  (0.4-2.2%) | 1.3%  (0.6-2.1%) | 1.065  (0.972, 1.162) | 0.179 |
|  |  |  |  |  |  |  |  |  |
| *STI Testing, Treatment, and Diagnosis* |  |  |  |  |  |  |  |  |
| Chlamydia testing in past 12 months | Hispanic | 23.6%  (19.4-27.7%) | 27.4%  (22.9-32.0%) | 30.8%  (26.9-34.7%) | 30.2%  (24.9-35.5%) | 29.4%  (24.8-33.1%) | 1.032  (1.000, 1.064) | 0.050 |
|  | NH Black | 46.3%  (41.9-50.7%) | 47.6%  (42.8-52.4%) | 53.4%  (48.2-58.6%) | 54.5%  (50.0-59.0%) | 53.0%  (46.8-59.1%) | **1.038**  **(1.008, 1.069)** | **0.014** |
|  | NH White | 23.1%  (20.5-25.7%) | 23.4%  (20.4-26.3%) | 26.3%  (23.8-28.8%) | 27.1%  (24.5-29.7%) | 24.9%  (21.8-28.0%) | 1.019  (0.997, 1.041) | 0.091 |
| STI testing in past 12 months† | Hispanic | NA | 34.2%  (28.7-39.6%) | 40.6%  (35.5-45.7%) | 39.6%  (35.1-44.1%) | 35.4%  (30.9-40.0%) | 1.005  (0.958, 1.055) | 0.836 |
|  | NH Black | NA | 55.3%  (51.1-59.5%) | 60.5%  (55.1-65.8%) | 60.5%  (55.7-65.3%) | 58.0%  (51.7-64.3%) | 1.017  (0.968, 1.068) | 0.500 |
|  | NH White | NA | 27.2%  (24.4-29.9%) | 29.8%  (27.4-32.2%) | 31.5%  (28.5-34.5%) | 30.5%  (27.3-33.7%) | 1.029  (0.997, 1.062) | 0.080 |
| STI treatment in past 12 months† | Hispanic | NA | 13.5%  (7.9-19.1%) | 13.2%  (9.7-16.8%) | 9.6%  (5.2-14.0%) | 14.7%  (9.6-19.8%) | 0.998  (0.899, 1.109) | 0.975 |
|  | NH Black | NA | 14.6%  (9.4-19.8%) | 14.2%  (10.1-18.3%) | 19.2%  (11.3-27.0%) | 12.8%  (9.6-16.0%) | 0.999  (0.922, 1.083) | 0.987 |
|  | NH White | NA | 12.7%  (8.6-16.9%) | 11.8%  (8.1-15.5%) | 9.6%  (5.5-13.6%) | 13.6%  (8.4-18.8%) | 1.002  (0.907, 1.108) | 0.964 |
| Gonorrhea diagnosis in past 12 months† | Hispanic | NA | 2.9%  (0.0-5.9%) | 1.5%  (0.4-2.5%) | 3.5%  (0.5-6.5%) | 2.3%  (0.2-4.3%) | 1.009  (0.808, 1.261) | 0.935 |
|  | NH Black | NA | 3.4%  (1.6-5.2%) | 1.9%  (0.8-2.8%) | 2.1%  (0.9-3.4%) | 4.6%  (2.2-7.0%) | 1.074  (0.919, 1.255) | 0.370 |
|  | NH White | NA | 1.2%  (0.4-1.9%) | 1.8%  (0.8-2.8%) | 1.2%  (0.5-2.0%) | 2.7%  (1.3-4.1%) | 1.128  (0.978, 1.301) | 0.098 |
| Chlamydia diagnosis in past 12 months† | Hispanic | NA | 6.2%  (2.3-10.1%) | 5.6% (3.5-7.7%) | 5.7%  (1.6-9.7%) | 3.6%  (1.8-5.5%) | 0.926  (0.809, 1.061) | 0.268 |
|  | NH Black | NA | 6.3%  (3.7-8.9%) | 5.7%  (2.9-8.5%) | 10.8%  (2.7-18.8%) | 6.2%  (3.6-8.9%) | 1.038  (0.938, 1.150) | 0.467 |
|  | NH White | NA | 3.0%  (1.5-4.4%) | 3.0%  (1.4-4.7%) | 4.1%  (1.7-6.5%) | 4.2%  (1.6-6.8%) | 1.073  (0.943, 1.221) | 0.284 |
| NH = Non-Hispanic. Bold indicates significance at <0.05 level. Non-Hispanic respondents reporting multiple races were categorized as “Another race/ethnicity” in NSFG public-use data and therefore excluded from these analyses.  *Estimates derived from linear regression (β). All others from logistic regression (odds ratios; OR). All models accounted for survey weights.  †Analysis includes 2011-13 through 2017-19 survey periods only. In the 2008-10 survey period, female respondents were not asked about STI testing other than chlamydia and were not asked about gonorrhea or chlamydia diagnoses unless they reported STI treatment in the past 12 months.  ^‡^Estimate based on numerator fewer than 5 cases or denominator fewer than 100 cases, and therefore does not meet standards of reliability or precision (Copen et al., 2016). | | | | | | | | |

| **Supplemental Table 8. Sexual behaviors and STI testing, treatment, and diagnosis among male respondents, stratified by race/ethnicity, in the 2008-10 to 2017-19 survey periods of the National Survey of Family Growth** | | | | | | | | |
| --- | --- | --- | --- | --- | --- | --- | --- | --- |
|  | **Race / Ethnicity** | **% or Mean (95% Confidence Interval)** | | | | | **Logistic or linear regression** | |
|  |  | **2008-10** | **2011-13** | **2013-15** | **2015-17** | **2017-19** | **OR or β (95%CI)** | **p-value** |
| *Sexual behaviors* |  |  |  |  |  |  |  |  |
| Ever had vaginal sex with female sex partner | Hispanic | 88.0%  (86.0-90.1%) | 86.8%  (84.3-89.3%) | 87.0%  (84.7-89.4%) | 86.5%  (83.7-89.2%) | 84.7%  (82.1-87.4%) | 0.973  (0.944, 1.002) | 0.071 |
|  | NH Black | 86.1%  (83.5-88.7%) | 90.6%  (87.7-93.4%) | 87.8%  (85.1-90.5%) | 85.4%  (82.0-88.8%) | 88.2%  (85.0-91.4%) | 1.000 (0.962, 1.040) | 0.994 |
|  | NH White | 85.3%  (83.8-86.7%) | 86.7%  (84.7-88.7%) | 86.8%  (85.1-88.6%) | 84.2%  (81.8-86.7%) | 84.7%  (82.4-87.1%) | 0.989  (0.968, 1.012) | 0.352 |
| Vaginal sex with female sex partner(s) in past 12 months | Hispanic | 89.9%  (86.5-93.4%) | 92.4%  (90.5-94.3%) | 88.9%  (85.8-92.0%) | 89.5%  (86.4-92.5%) | 89.1%  (86.2-92.1%) | 0.979  (0.931, 1.029) | 0.402 |
|  | NH Black | 92.9%  (91.0-94.8%) | 94.3%  (92.4-96.2%) | 91.1%  (87.6-94.6%) | 89.2%  (85.8-92.6%) | 90.4%  (87.3-93.6%) | **0.944**  **(0.900, 0.990)** | **0.018** |
|  | NH White | 90.9%  (89.6-92.3%) | 89.8%  (88.3-91.4%) | 91.8%  (90.3-93.2%) | 91.4%  (89.7-93.1%) | 90.9%  (88.7-93.2%) | 1.008 (0.977, 1.039) | 0.624 |
| Number of female sex partners in past 12 months* | Hispanic | 1.43  (1.33-1.52) | 1.38  (1.30-1.45) | 1.43  (1.31-1.55) | 1.29  (1.23-1.35) | 1.36  (1.25-1.46) | -0.010  (-0.024, 0.003) | 0.135 |
|  | NH Black | 1.87  (1.71-2.04) | 1.82  (1.70-1.94) | 1.80  (1.64-1.96) | 1.86  (1.68-2.04) | 1.66  (1.48-1.84) | -0.017  (-0.042, 0.007) | 0.168 |
|  | NH White | 1.39  (1.34-1.44) | 1.41  (1.34-1.49) | 1.36  (1.30-1.43) | 1.34  (1.27-1.41) | 1.29  (1.25-1.34) | **-0.011**  **(-0.019, -0.004)** | **0.004** |
| Condom use at last vaginal sex | Hispanic | 37.9%  (33.2-42.6%) | 41.8%  (36.3-47.3%) | 35.3%  (29.8-40.7%) | 36.2%  (31.8-40.5%) | 33.9%  (29.5-38.3%) | 0.975  (0.949, 1.003) | 0.080 |
|  | NH Black | 45.6%  (42.0-49.1%) | 46.7%  (41.7-51.6%) | 44.7%  (39.0-50.4%) | 36.8%  (30.1-43.5%) | 40.4%  (35.4-45.4%) | **0.965**  **(0.938, 0.992)** | **0.011** |
|  | NH White | 31.3%  (29.3-33.4%) | 32.4%  (29.8-35.0%) | 30.8%  (27.4-34.2%) | 28.9%  (25.1-32.8%) | 27.6%  (24.0-31.2%) | **0.978**  **(0.959, 0.998)** | **0.032** |
| ≥1 vaginal sex act in past 4 weeks | Hispanic | 80.1%  (75.1-85.0%) | 83.9%  (81.1-86.6%) | 82.0%  (78.8-85.3%) | 79.9%  (74.9-84.9%) | 78.5%  (74.1-82.8%) | 0.981  (0.938, 1.025) | 0.388 |
|  | NH Black | 83.7%  (79.9-87.5%) | 80.8%  (76.1-85.6%) | 79.3%  (75.6-83.1%) | 83.7%  (78.8-88.6%) | 77.0%  (71.9-82.1%) | 0.968  (0.927, 1.010) | 0.137 |
|  | NH White | 81.9%  (79.3-84.5%) | 80.7%  (78.3-83.0%) | 82.2%  (79.9-84.5%) | 81.1%  (78.3-83.8%) | 78.9%  (75.7-82.1%) | 0.984  (0.959, 1.011) | 0.236 |
| Number of vaginal sex acts in past 4 weeks* | Hispanic | 6.79  (5.27-8.31) | 6.37  (5.83-6.91) | 6.42  (5.80-7.03) | 6.21  (5.29-7.14) | 5.65  (4.88-6.43) | -0.110  (-0.276, 0.055) | 0.191 |
|  | NH Black | 5.24  (4.61-5.88) | 6.14  (5.32-6.97) | 5.99  (5.06-6.92) | 5.68  (4.75-6.62) | 4.55  (3.76-5.34) | -0.072  (-0.183, 0.039) | 0.207 |
|  | NH White | 5.40  (5.01-5.80) | 5.87  (5.42-6.32) | 5.99  (5.62-6.36) | 5.56  (5.06-6.07) | 5.04  (4.66-5.42) | -0.035  (-0.093, 0.023) | 0.241 |
| Proportion of condom-protected vaginal sex acts in past 4 weeks* | Hispanic | 0.308  (0.264-0.353) | 0.306  (0.256-0.356) | 0.258  (0.202-0.314) | 0.301  (0.265-0.338) | 0.267  (0.216-0.318) | -0.004  (-0.011, 0.003) | 0.242 |
|  | NH Black | 0.404  (0.363-0.445) | 0.350  (0.299-0.402) | 0.362  (0.302-0.422) | 0.296  (0.242-0.351) | 0.349  (0.288-0.409) | **-0.008**  **(-0.015, -0.001)** | **0.032** |
|  | NH White | 0.266  (0.239-0.293) | 0.276  (0.242-0.309) | 0.266  (0.231-0.301) | 0.211  (0.185-0.237) | 0.226  (0.191-0.261) | **-0.006**  **(-0.011, -0.002)** | **0.006** |
| Ever had oral or anal sex with another man | Hispanic | 2.6%  (1.5-3.7%) | 4.1%  (2.1-6.2%) | 2.2%  (1.3-3.1%) | 4.7%  (2.2-7.2%) | 5.0%  (2.4-7.5%) | 1.071  (0.990, 1.158) | 0.087 |
|  | NH Black | 3.0%  (2.1-4.0%) | 3.3%  (0.6-6.1%) | 2.1%  (0.9-3.3%) | 3.8%  (1.1-6.6%) | 1.8%  (0.8-2.7%) | 0.967  (0.896, 1.044) | 0.391 |
|  | NH White | 3.9%  (2.8-5.0%) | 4.7%  (3.5-6.0%) | 4.1%  (2.8-5.5%) | 4.5%  (2.8-6.1%) | 5.6%  (4.4-6.8%) | 1.034  (0.994, 1.076) | 0.100 |
| Racial/ethnic homophily among up to 3 current partners with whom vaginal sex occurred most recently | Hispanic | 79.9%  (76.1-83.7%) | 75.3%  (69.5-81.0%) | 73.1%  (67.5-78.6%) | 72.9%  (66.3-79.5%) | 75.3%  (69.5-81.1%) | 0.971  (0.933, 1.011) | 0.151 |
|  | NH Black | 79.5%  (74.7-84.2%) | 76.5%  (71.7-81.2% | 72.7%  (66.2-79.1%) | 74.9%  (68.4-81.3%) | 65.9%  (58.7-73.2%) | **0.935**  **(0.895, 0.978)** | **0.003** |
|  | NH White | 89.6%  (86.8-92.4%) | 87.5%  (84.9-90.2%) | 88.9%  (86.2-91.5%) | 83.1%  (79.6-86.7%) | 86.2%  (83.3-89.1%) | **0.954**  **(0.919, 0.992)** | **0.017** |
| Concurrency (≥2 current partners at time of survey) | Hispanic | 2.1%  (1.0-3.3%) | 2.4%  (1.3-3.6%) | 2.1%  (0.7-3.6%) | 1.7%  (0.7-2.8%) | 2.0%  (0.9-3.1%) | 0.980  (0.908, 1.058) | 0.605 |
|  | NH Black | 8.2%  (5.6-10.7%) | 4.2%  (2.9-5.4%) | 5.0%  (2.5-7.4%) | 7.1%  (2.3-11.9%) | 2.8%  (1.3-4.2%) | 0.929  (0.861, 1.002) | 0.055 |
|  | NH White | 2.1%  (1.1-3.2%) | 1.8%  (1.0-2.6%) | 2.3%  (1.3-3.4%) | 1.2%  (0.6-1.7%) | 1.0%  (0.5-1.5%) | **0.925**  **(0.867, 0.987)** | **0.018** |
|  |  |  |  |  |  |  |  |  |
| *STI Testing, Treatment, and Diagnosis* |  |  |  |  |  |  |  |  |
| STI testing in past 12 months | Hispanic | 17.4%  (14.3-20.5%) | 18.9%  (15.9-21.8%) | 16.9%  (13.4-20.4%) | 21.8%  (16.7-27.0%) | 15.4%  (12.0-18.7%) | 0.997  (0.964, 1.032) | 0.881 |
|  | NH Black | 38.2%  (34.4-42.0%) | 33.9%  (28.8-39.0%) | 37.2%  (29.2-45.2%) | 32.5%  (25.4-39.6%) | 32.5%  (26.4-38.5%) | 0.974  (0.942, 1.007) | 0.119 |
|  | NH White | 12.8%  (10.3-15.4%) | 13.3%  (10.6-16.1%) | 12.0%  (10.3-13.7%) | 12.8%  (10.1-15.5%) | 10.1%  (8.6-11.7%) | 0.977  (0.948, 1.007) | 0.134 |
| STI treatment in past 12 months | Hispanic | 14.7%  (7.7-21.7%) | 13.7%  (6.1-21.3%) | 10.6%  (4.3-16.8%) | 13.0%  (0.8-25.2%) | 11.3%  (5.5-17.2%) | 0.979  (0.836, 1.146) | 0.789 |
|  | NH Black | 19.9%  (13.5-26.4%) | 14.0%  (9.1-18.9%) | 21.2%  (14.0-28.4%) | 20.3%  (8.5-32.2%) | 11.8%  (5.5-18.1%) | 0.973  (0.875, 1.081) | 0.605 |
|  | NH White | 14.6%  (8.0-21.2%) | 7.9%  (4.6-11.2%) | 10.4%  (5.0-15.8%) | 9.7% (5.0-14.3%) | 8.5%  (4.6-12.4%) | 1.011  (0.913, 1.120) | 0.829 |
| Gonorrhea diagnosis in past 12 months† | Hispanic | NA | 1.7%  (0.3-3.2%) | 1.0%  (0.0-2.3%)^‡^ | 0.4%  (0.0-1.0%)^‡^ | 1.5%  (0.0-3.3%)^‡^ | 0.929  (0.679, 1.272) | 0.646 |
|  | NH Black | NA | 3.7%  (1.9-5.6%) | 3.6%  (1.5-5.7%) | 5.3%  (0.7-10.0%) | 2.9%  (0.4-5.5%) | 0.990  (0.854, 1.148) | 0.897 |
|  | NH White | NA | 2.1%  (0.5-3.7%) | 2.1%  (0.1-4.0%) | 2.6%  (0.0-5.5%) | 2.0%  (0.0-4.0%) | 1.009  (0.826, 1.232) | 0.928 |
| Chlamydia diagnosis in past 12 months† | Hispanic | NA | 2.9%  (1.0-4.8%) | 1.4%  (0.0-2.8%)^‡^ | 0.6%  (0.0-1.6%)^‡^ | 4.1%  (0.5-7.8%) | 1.041  (0.793, 1.366) | 0.771 |
|  | NH Black | NA | 5.5%  (2.4-8.5%) | 10.7%  (5.9-15.5%) | 12.8%  (1.9-23.6%) | 4.2%  (0.0-9.1%) | 0.988  (0.851, 1.146) | 0.870 |
|  | NH White | NA | 3.2%  (1.4-5.0%) | 2.8%  (0.7-5.0%) | 4.7%  (1.2-8.2%) | 2.6%  (0.4-4.8%) | 1.008  (0.868, 1.172) | 0.912 |
| NH = Non-Hispanic. Bold indicates significance at <0.05 level. Non-Hispanic respondents reporting multiple races were categorized as “Another race/ethnicity” in NSFG public-use data and therefore excluded from these analyses.  *Estimates derived from linear regression (β). All others from logistic regression (odds ratios; OR). All models accounted for survey weights.  †Analysis includes 2011-13 through 2017-19 survey periods only. In the 2008-10 survey period, male respondents were not asked about gonorrhea or chlamydia diagnoses unless they reported STI treatment in the past 12 months.  ^‡^Estimate based on numerator fewer than 5 cases or denominator fewer than 100 cases, and therefore does not meet standards of reliability or precision (Copen et al., 2016). | | | | | | | | |

**REFERENCE**

Copen, C. E., Chandra, A., & Febo-Vazquez, I. (2016). Sexual Behavior, Sexual Attraction, and Sexual Orientation Among Adults Aged 18-44 in the United States: Data From the 2011-2013 National Survey of Family Growth. *Natl Health Stat Report*(88), 1-14.
